# Pectin and Kaolin as Adjunctive Metabolic Therapy for Maintaining Remission in Ulcerative Colitis: Biological Rationale, Current Evidence and Future Clinical Perspectives

**DOI:** 10.3390/nu18172822

**Published:** 2026-08-28

**Authors:** John K. Triantafillidis, Andreas Panayiotou, Apostolos E. Papalois

**Affiliations:** 1 Hellenic Society for Gastrointestinal Oncology, 354 Iera Odos Str., 12461 Haidari, Greece; jktrian@gmail.com; 2 Academic Hub, Mediterranean Hospital of Cyprus, 9 Stygos Str., Limassol 3117, Cyprus; dr.panayiotou@medihospital.com.cy

**Keywords:** ulcerative colitis, pectin, kaolin, short-chain fatty acids, butyrate, gut microbiome, intestinal barrier, metabolic therapy, remission maintenance, adjunctive therapy

## Abstract

**Background:** Ulcerative colitis (UC) is a chronic relapsing inflammatory bowel disease in which long-term maintenance of remission remains a major therapeutic challenge. Increasing evidence indicates that disruption of host–microbiome metabolic interactions, impaired production of short-chain fatty acids (SCFAs), defective epithelial energy metabolism, and intestinal barrier dysfunction contribute to disease relapse. These observations have generated interest in adjunctive metabolic strategies aimed at restoring luminal homeostasis rather than directly suppressing inflammation. **Objective:** This narrative review examines the biological rationale for combining pectin, a fermentable dietary fiber that promotes endogenous SCFA production, with kaolin, naturally occurring adsorbent clay, as adjunctive metabolic therapy for maintaining remission in UC. We summarize current mechanistic, experimental, and clinical evidence and discuss future translational research priorities. **Evidence:** Experimental studies demonstrate that pectin fermentation enhances the production of butyrate and other SCFAs, thereby improving epithelial energy metabolism, strengthening intestinal barrier integrity, and modulating mucosal immune responses. Kaolin possesses adsorptive and barrier-protective properties that may reduce luminal exposure to potentially harmful microbial products while improving stool consistency. Although direct clinical evidence supporting the combined use of pectin and kaolin in UC is currently lacking, indirect evidence derived from microbiome research, SCFA biology, dietary intervention studies, and experimental models provides a biologically plausible foundation for further investigation. **Perspective:** We propose that restoration of luminal metabolic homeostasis through complementary modulation of microbial fermentation and luminal environmental stabilization may represent a novel adjunctive approach for reducing relapse risk in selected patients with UC receiving conventional maintenance therapy. A conceptual framework for future randomized clinical trials incorporating clinical, endoscopic, microbiome, metabolomic, and biomarker outcomes is presented. **Conclusions:** Current evidence supports the biological plausibility of SCFA-directed metabolic interventions in UC but remains insufficient to justify routine clinical application of pectin–kaolin therapy. Well-designed randomized controlled trials are required to determine efficacy, safety, optimal patient selection, and mechanisms of action. By integrating advances in microbiome science, intestinal metabolism, and mucosal immunology, this review provides a translational framework for the development of inexpensive, microbiome-oriented adjunctive therapies for maintenance of remission in UC.

## 1. Introduction

Ulcerative colitis (UC) is a chronic inflammatory disorder of the colonic mucosa characterized by relapsing–remitting episodes of inflammation and disruption of the epithelial barrier. Its incidence and prevalence have been increasing worldwide, including in underdeveloped countries, with a substantial impact on patients’ quality of life and healthcare systems. Despite advances in pharmacological therapy, a significant proportion of patients experience frequent relapses, underscoring the need for complementary strategies that target underlying pathogenic mechanisms [1,2].

UC pathogenesis involves not only immune dysregulation but also ecological and metabolic dysfunction within the gut microbiome. Emerging evidence implicates gut microbiota dysbiosis as a central factor in the pathogenesis of UC. Compared with healthy individuals, patients with UC display reduced diversity of commensal bacteria, depletion of butyrate-producing species such as *Faecalibacterium prausnitzii* and *Roseburia hominis*, and expansion of potentially pro-inflammatory taxa [3,4,5]. These alterations are associated with impaired production of short-chain fatty acids (SCFAs), particularly butyrate, which are critical for maintaining intestinal epithelial integrity, modulating immune responses, and providing energy substrates for colonocytes [4,6,7,8,9,10]. SCFAs regulate mucosal immunity through several mechanisms, including induction of regulatory T cells (Tregs), inhibition of histone deacetylases, suppression of pro-inflammatory signaling pathways such as NF-κB, and enhancement of tight-junction protein expression [8,9,11,12]. Therefore, restoring microbial metabolic competence may represent a complementary, non-immunosuppressive strategy to prolong remission [5].

Dietary interventions, particularly prebiotic supplementation, have been proposed to restore SCFA production and promote microbial homeostasis. Prebiotics such as pectin are fermented by colonic microbiota, resulting in the production of acetate, propionate, and butyrate [13,14,15,16], which collectively contribute to anti-inflammatory effects and epithelial barrier reinforcement. Preclinical and clinical studies have demonstrated that increasing SCFA availability can reduce mucosal inflammation, improve epithelial barrier function, and decrease relapse risk in patients with UC.

Another potential adjunctive strategy is the use of kaolin as adsorptive clay, traditionally used to manage diarrhea. Kaolin may confer mucosal protection by binding luminal toxins and reducing their interaction with the colonic epithelium [9,11], potentially mitigating inflammatory triggers. While kaolin has not been systematically studied in UC, its physicochemical properties suggest a plausible mechanism to enhance barrier integrity and to synergize with microbiota-derived metabolites. Importantly, combination strategies that integrate prebiotics or kaolin with standard pharmacotherapy, such as mesalazine, may provide additive or synergistic benefits. Mesalazine exerts anti-inflammatory effects through inhibition of cyclooxygenase pathways, suppression of NF-κB, and modulation of local cytokine production [1,2]. Augmenting mesalazine therapy with SCFA-producing prebiotics and kaolin could theoretically enhance mucosal protection, restore microbial balance, and reduce relapse frequency, providing a rationale for future exploratory clinical trials. To date, however, there is limited human evidence for pectin [17] and no clinical studies directly evaluating kaolin supplementation in UC patients [11,18,19], highlighting a critical gap in the literature. Preclinical data on SCFA-mediated immune regulation, microbiota modulation, and epithelial protection support the biological plausibility of this approach, yet rigorous clinical validation is required.

This review aims to synthesize current mechanistic evidence, elucidate potential pathways by which prebiotics and kaolin could modulate disease course, and propose directions for future research, including combination strategies with standard therapy.

## 2. Microbial Metabolic Dysfunction in UC

Microbial metabolic dysfunction has emerged as a central component of UC pathogenesis, extending the concept of intestinal dysbiosis beyond simple alterations in microbial composition. Rather than representing only taxonomic shifts, dysbiosis in UC is increasingly recognized as a functional disorder characterized by impaired microbial metabolic activity, depletion of beneficial metabolites, accumulation of pro-inflammatory compounds, and disruption of host–microbiome metabolic crosstalk [5,20,21]. These alterations compromise epithelial energy metabolism, weaken intestinal barrier integrity, promote immune activation, and contribute to persistent mucosal inflammation and disease relapse.

Among the metabolic abnormalities identified in UC, reduced production of SCFAs, particularly butyrate, together with altered bile acid metabolism, increased succinate generation, impaired tryptophan metabolism, and enhanced lipopolysaccharide biosynthesis constitute the most consistently reported findings [6,10,22,23]. These metabolic disturbances create a luminal environment that favors chronic inflammation while limiting epithelial repair and restoration of mucosal homeostasis.

Increasing evidence suggests that functional metabolic alterations may predict disease behavior more accurately than microbial composition alone. Advances in metagenomics, metatranscriptomics, metabolomics, and other multi-omics technologies have demonstrated that microbial metabolic capacity is often profoundly altered even when taxonomic differences appear modest [24,25,26,27]. Consequently, restoration of microbial metabolic function has emerged as an attractive therapeutic target that complements conventional anti-inflammatory treatment and provides the biological rationale for microbiome-directed metabolic interventions.

The principal microbial-derived metabolites currently implicated in UC pathophysiology, together with their major producing microorganisms and biological functions, are summarized in Table 1.

**Figure 1:**
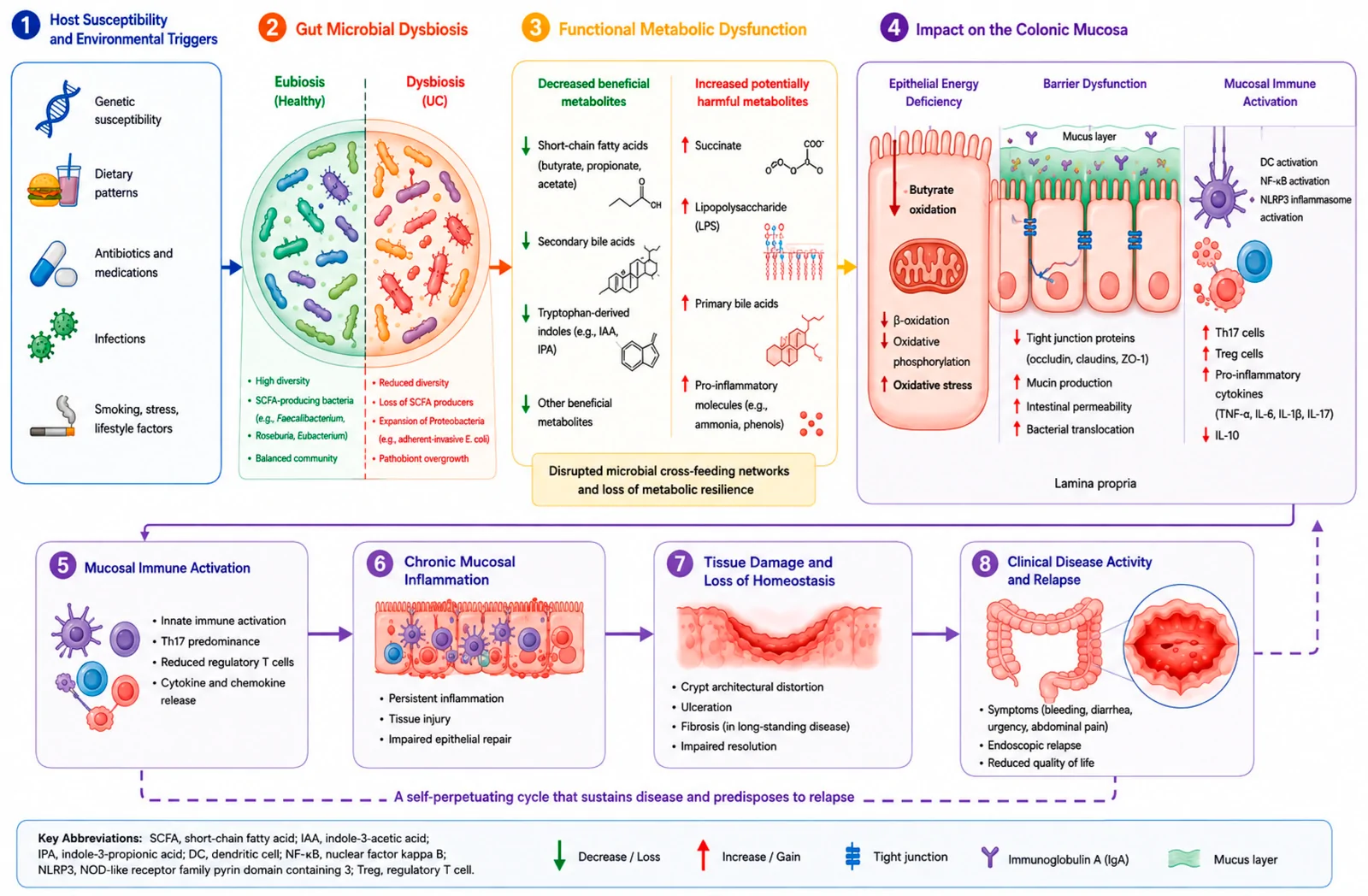
Metabolic dysfunction as the central pathogenic pathway in UC. Genetic susceptibility and environmental factors promote gut microbial dysbiosis, leading to depletion of SCFA-producing bacteria and widespread alterations in microbial metabolism. Reduced production of beneficial microbial metabolites together with accumulation of pro-inflammatory metabolites impairs colonocyte mitochondrial metabolism, disrupts epithelial barrier integrity, activates mucosal immune responses, and ultimately drives chronic intestinal inflammation and disease relapse. This metabolic framework provides the biological rationale for the microbiome-directed therapeutic strategies discussed in subsequent sections of this review.

Figure 1 displays a schematic representation of the metabolic dysfunction that appears in UC.

### 2.1. Dysbiosis Beyond Taxonomy

The traditional concept of dysbiosis in UC has largely focused on alterations in microbial composition. However, accumulating evidence indicates that taxonomic changes alone provide only a partial explanation of disease pathogenesis. Advances in metagenomics, metatranscriptomics, metabolomics, and systems biology have demonstrated that functional alterations within the intestinal microbiome may be more relevant than simple shifts in bacterial abundance. Consequently, dysbiosis is increasingly regarded as a state of impaired microbial metabolic function rather than merely an altered microbial community structure.

Patients with UC typically exhibit reduced microbial diversity together with depletion of obligate anaerobic, SCFA-producing bacteria, particularly members of the Firmicutes phylum, including *Faecalibacterium prausnitzii*, *Roseburia hominis*, *Eubacterium rectale*, and other butyrate-producing species [3,4,5,29]. Conversely, facultative anaerobic organisms belonging to Proteobacteria, including adherent-invasive *Escherichia coli*, together with *Enterococcus* spp. and other potentially pro-inflammatory taxa, frequently become enriched. Although these compositional changes are consistently observed across multiple cohorts, considerable inter-individual variability limits their value as independent disease biomarkers [30]. More importantly, functional analyses have revealed profound alterations in microbial metabolic capacity. Genes involved in complex carbohydrate degradation, butyrate biosynthesis, secondary bile acid transformation, and tryptophan metabolism are consistently reduced [5,20,21], whereas pathways associated with oxidative stress, nitrate respiration, lipopolysaccharide biosynthesis, and production of pro-inflammatory metabolites are frequently enriched. Disruption of microbial cross-feeding networks further compromises the conversion of primary fermentation products into butyrate [9,20,21], thereby amplifying metabolic insufficiency despite the persistence of certain bacterial taxa.

These functional abnormalities have important biological consequences for the colonic mucosa. Reduced production of SCFAs limits the principal energy supply of colonocytes [8,10,31], impairs mitochondrial oxidative metabolism, weakens epithelial barrier integrity, and promotes activation of innate and adaptive immune pathways. Simultaneously, accumulation of metabolites such as succinate and alterations in bile acid and tryptophan metabolism [8,9,20,32] further contribute to epithelial dysfunction and chronic mucosal inflammation. Thus, the transition from taxonomic dysbiosis to metabolic dysfunction represents a critical step linking alterations in the gut microbiome to the development and persistence of intestinal inflammation.

Therefore, current evidence supports the concept that microbial function is more informative than microbial composition alone [24,25,26,27,29]. This paradigm shift provides the biological foundation for therapeutic strategies aimed at restoring microbial metabolic activity through dietary modulation, prebiotics, microbiome-directed therapies, and other metabolically targeted interventions rather than simply attempting to alter bacterial composition.

### 2.2. SCFAs: Central Mediators of Intestinal Homeostasis

SCFAs are the principal metabolic products generated by anaerobic bacterial fermentation of non-digestible dietary carbohydrates in the colon and constitute key mediators of host–microbiome interactions. Acetate, propionate, and butyrate account for approximately 95% of total luminal SCFAs [7,20,28], with acetate representing nearly 60%, propionate approximately 20%, and butyrate approximately 20%. Although all three metabolites contribute to intestinal homeostasis, butyrate has emerged as the most biologically relevant SCFA in UC because it serves as the primary oxidative substrate for colonocytes [32,33,34] while simultaneously regulating epithelial barrier integrity, mucosal immunity, and inflammatory signaling [20,21,28,33,35].

Βeyond their role as microbial fermentation products, SCFAs function as pleiotropic signaling molecules linking dietary intake, microbial metabolism, epithelial physiology, and immune regulation. After their production within the colonic lumen, SCFAs are absorbed through specific epithelial transporters, including monocarboxylate transporter-1 (MCT1/SLC16A1) and sodium-coupled monocarboxylate transporter-1 (SMCT1/SLC5A8), and subsequently participate in multiple intracellular metabolic and signaling pathways [33,34]. In healthy individuals, butyrate supplies up to 70% of the energy requirements of colonocytes, thereby maintaining mitochondrial oxidative phosphorylation, physiological epithelial hypoxia, and mucosal barrier integrity. These physiological mechanisms are substantially impaired in UC. Active disease is consistently associated with reduced fecal concentrations of total SCFAs [13,32,36], depletion of butyrate-producing bacterial species, impaired epithelial uptake of butyrate, and diminished β-oxidation within colonocytes [33,34]. Consequently, epithelial cells undergo metabolic reprogramming characterized by reduced oxidative phosphorylation, increased glycolysis, mitochondrial dysfunction, oxidative stress, and impaired mucosal repair. These metabolic alterations contribute directly to epithelial barrier disruption and facilitate continuous exposure of the mucosal immune system to luminal microbial products.

SCFAs also exert broad immunoregulatory effects through both receptor-dependent and receptor-independent mechanisms. Binding to G protein-coupled receptors—including FFAR2 (GPR43), FFAR3 (GPR41), and HCAR2 (GPR109A)—modulates epithelial signaling, cytokine production, inflammasome activation, and leukocyte recruitment [20,28,33]. Simultaneously, intracellular inhibition of histone deacetylases (HDACs), particularly by butyrate, regulates gene transcription involved in immune tolerance, epithelial differentiation, and inflammatory responses [20,21,33]. These complementary mechanisms promote expansion of FoxP3^+^ regulatory T cells, suppress Th17-mediated inflammation, inhibit NF-κB activation, reduce production of pro-inflammatory cytokines, and enhance IL-10-mediated anti-inflammatory signaling. In parallel, SCFAs reinforce multiple components of the intestinal barrier [15,21,33,37]. Butyrate stimulates epithelial proliferation, increases expression of tight-junction proteins including occludin, claudins, and zonula occludens-1, promotes mucus secretion by goblet cells, enhances antimicrobial peptide production, and supports physiological activation of the NLRP3 inflammasome with subsequent IL-18 release, thereby preserving epithelial integrity and reducing bacterial translocation [15,21,33,37]. Butyrate may additionally reinforce the mucus barrier through macrophage-dependent WNT/ERK signaling, promoting goblet-cell differentiation and MUC2 expression [37]. These coordinated actions illustrate that SCFAs act not merely as metabolic substrates but as important regulators of epithelial homeostasis.

Multi-omics studies integrating metagenomics, metatranscriptomics, metabolomics, and transcriptomics have further expanded our understanding of SCFA biology by demonstrating extensive interactions between microbial metabolism and host signaling pathways. Alterations in SCFA availability influence bile acid metabolism through FXR and TGR5 signaling, modulate tryptophan-derived aryl hydrocarbon receptor (AhR) ligands [8,9,11,20], regulate mitochondrial function, and interact with multiple metabolic pathways involved in epithelial regeneration and immune homeostasis. These findings support the concept that SCFAs occupy a central position within an integrated host–microbiome metabolic network rather than functioning as isolated microbial metabolites.

Although reduced SCFA concentrations represent one of the most reproducible metabolic abnormalities observed in UC, quantitative deficiencies alone probably do not fully explain disease pathogenesis. Increasing evidence suggests that impaired epithelial utilization of SCFAs and disruption of downstream signaling pathways may be equally important determinants of disease activity. Moreover, experimental human physiomimetic models indicate that the biological effects of SCFAs may be context-dependent, with beneficial effects on innate inflammatory responses but potentially adverse effects during acute T-cell-mediated inflammation [38]. Thus, restoration of microbial metabolic competence, enhancement of endogenous SCFA production, and recovery of epithelial responsiveness collectively represent biologically plausible therapeutic targets that may complement conventional anti-inflammatory treatment [10,23,24].

**Figure 2:**
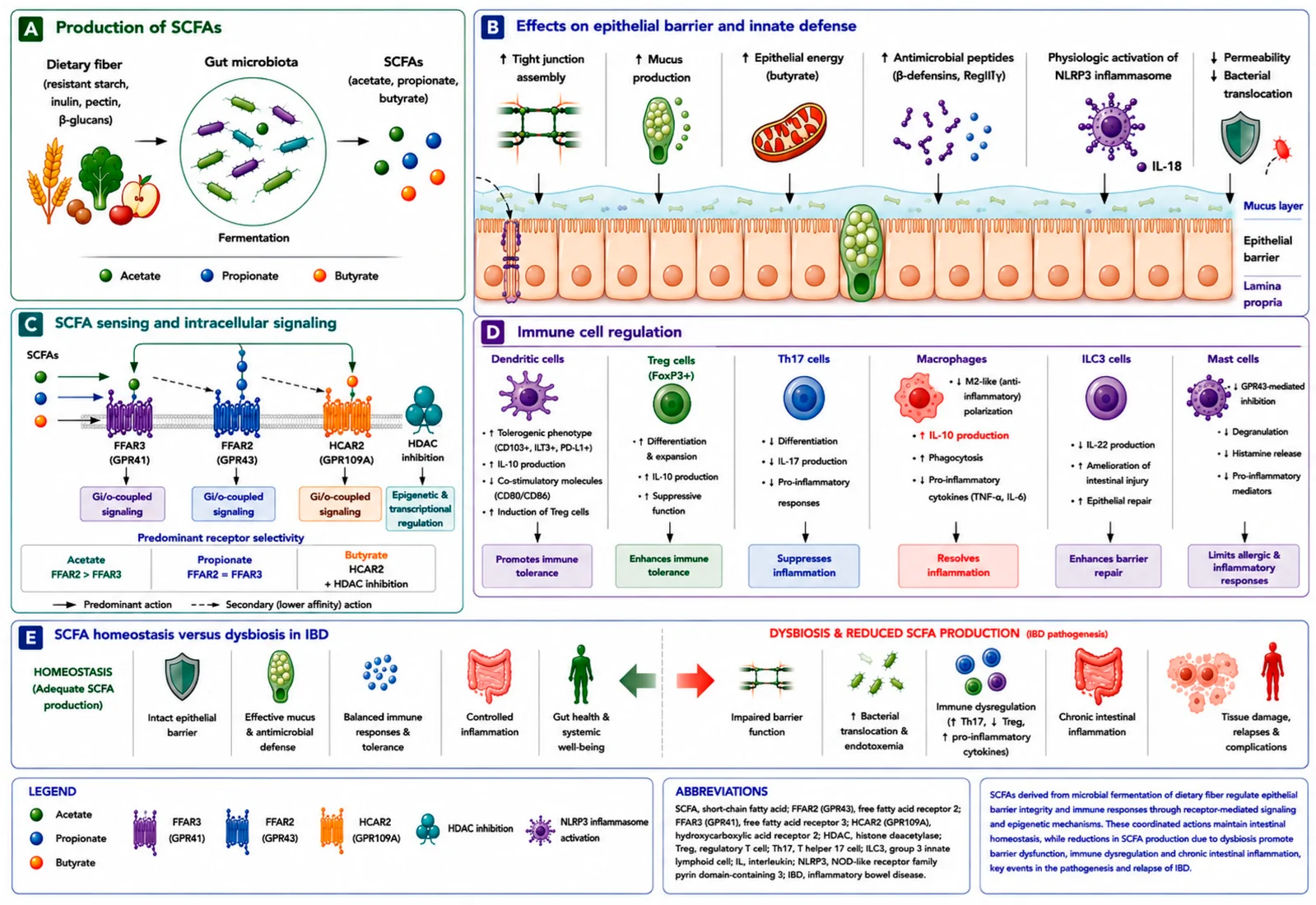
SCFA-mediated immune and barrier regulation pathway in intestinal homeostasis and IBD. This schematic summarizes the central role of SCFAs in maintaining intestinal homeostasis and regulating mucosal immunity. (**A**) Dietary fiber is fermented by the gut microbiota to generate the major SCFAs—acetate, propionate, and butyrate. (**B**) SCFAs reinforce epithelial barrier integrity by enhancing tight-junction assembly, stimulating mucus secretion, providing metabolic fuel for colonocytes (particularly butyrate), inducing antimicrobial peptide production (e.g., β-defensins and RegIIIγ), and supporting physiological activation of the NLRP3 inflammasome with subsequent IL-18 production, thereby reducing intestinal permeability and bacterial translocation. (**C**) SCFAs signal through the G protein-coupled receptors FFAR2 (GPR43), FFAR3 (GPR41), and HCAR2 (GPR109A), as well as through histone deacetylase (HDAC) inhibition. The figure also illustrates the predominant receptor selectivity of individual SCFAs, with acetate acting mainly through FFAR2, propionate preferentially activating FFAR2 and FFAR3, and butyrate signaling predominantly through HCAR2 while serving as the principal endogenous HDAC inhibitor. (**D**) Downstream immune regulation includes induction of tolerogenic dendritic cells, expansion of FoxP3^+^ regulatory T (Treg) cells, suppression of Th17-mediated responses, polarization of macrophages toward an IL-10-producing M2-like phenotype, stimulation of IL-22 production by group 3 innate lymphoid cells (ILC3s), and inhibition of mast cell activation. (**E**) Adequate SCFA production preserves epithelial integrity, immune tolerance, and intestinal homeostasis, whereas dysbiosis-associated reductions in SCFA production contribute to barrier dysfunction, bacterial translocation, immune dysregulation, chronic intestinal inflammation, and tissue injury, thereby representing a key mechanistic pathway in the pathogenesis and relapse of IBD. Abbreviations: SCFA, short-chain fatty acid; FFAR2 (GPR43), free fatty acid receptor 2; FFAR3 (GPR41), free fatty acid receptor 3; HCAR2 (GPR109A), hydroxycarboxylic acid receptor 2; HDAC, histone deacetylase; Treg, regulatory T cell; Th17, T helper 17 cell; ILC3, group 3 innate lymphoid cell; IL, interleukin; NLRP3, NOD-like receptor family pyrin domain-containing 3; IBD, inflammatory bowel disease.

Figure 2 summarizes the principal mechanisms through which SCFAs regulate epithelial metabolism, intestinal barrier function, and mucosal immune homeostasis, thereby providing the mechanistic framework supporting microbiome-directed metabolic therapies in UC.

#### Molecular Mechanisms of SCFA Action

Among the biological activities of SCFAs, their capacity to regulate gene expression through epigenetic mechanisms has emerged as one of the most important links between the intestinal microbiota and host immunity. Unlike conventional cytokine-mediated signaling pathways, epigenetic regulation modifies cellular responses without altering the underlying DNA sequence, thereby providing a dynamic interface through which microbial metabolites influence epithelial homeostasis and immune function. Butyrate is the most potent endogenous epigenetic regulator among the major SCFAs. After entering colonocytes through specific monocarboxylate transporters, intracellular butyrate inhibits class I and class II histone deacetylases (HDACs), resulting in increased histone acetylation and enhanced chromatin accessibility. This process modifies transcription of numerous genes involved in epithelial differentiation, oxidative metabolism, barrier maintenance, antimicrobial defense, and immune regulation.

One of the best-characterized consequences of HDAC inhibition is the promotion of immune tolerance through induction and stabilization of FoxP3^+^ regulatory T (Treg) cells. Increased histone acetylation within the *FOXP3* locus enhances transcriptional activity, leading to expansion of anti-inflammatory Treg populations and increased production of IL-10. Simultaneously, butyrate suppresses differentiation of pro-inflammatory Th1 and Th17 lymphocytes while attenuating production of TNF-α, IL-6, IL-17, and other inflammatory mediators that contribute to chronic intestinal inflammation [28].

Epigenetic regulation by SCFAs also extends to the intestinal epithelium. HDAC inhibition enhances expression of tight-junction proteins, including occludin, claudin-1, and zonula occludens-1, stimulates goblet-cell differentiation and mucin synthesis, promotes antimicrobial peptide production, and improves epithelial resistance to inflammatory injury [21]. These coordinated responses reinforce barrier integrity while simultaneously reducing bacterial translocation and activation of mucosal immune pathways.

In addition to HDAC inhibition, emerging evidence indicates that SCFAs interact with multiple epigenetic and metabolic regulatory networks, including AMP-activated protein kinase (AMPK), hypoxia-inducible factor-1α (HIF-1α), peroxisome proliferator-activated receptor-γ (PPARγ), and pathways controlling mitochondrial biogenesis and oxidative phosphorylation. Through these interconnected mechanisms, microbial metabolites integrate cellular metabolism with transcriptional regulation, thereby coordinating epithelial repair, immune homeostasis, and tissue regeneration. Accumulating evidence suggests that impairment of these epigenetic mechanisms contributes to UC pathogenesis. Reduced microbial production of butyrate, diminished epithelial uptake, and defective intracellular metabolism collectively limit HDAC inhibition and other downstream signaling events, thereby weakening barrier integrity and promoting persistent mucosal inflammation. Restoration of endogenous SCFA production may therefore represent not only a metabolic intervention but also an indirect epigenetic therapeutic strategy capable of re-establishing immune tolerance and epithelial homeostasis. Taken together, these findings establish SCFAs as central regulators of intestinal homeostasis rather than simple microbial fermentation products. Their coordinated effects on epithelial metabolism, barrier integrity, immune regulation, oxidative balance, and microbial ecology provide the biological rationale for therapeutic strategies aimed at restoring endogenous SCFA production. The following section, therefore, examines the principal nutritional approaches currently available to re-establish SCFA generation within the colonic ecosystem, with particular emphasis on microbiome-directed interventions.

Table 2 summarizes the principal molecular mechanisms through which SCFAs regulate epithelial metabolism, immune responses, barrier integrity, and epigenetic signaling in UC.

### 2.3. Strategies for Restoring Microbial SCFA Production

Experimental and clinical evidence consistently indicates that impaired production and utilization of SCFAs, particularly butyrate, represent an important metabolic abnormality in UC. Although alterations in microbial composition are well recognized, quantitative and functional deficiencies of SCFAs may provide a more direct reflection of the disrupted metabolic relationship between the intestinal microbiota and the host. Metabolomic studies have demonstrated that patients with active UC may exhibit reduced fecal concentrations of butyrate, acetate, and propionate compared with healthy individuals, with butyrate showing a particularly consistent reduction [23,24,25]. These changes parallel the depletion of major butyrate-producing bacteria, including *Faecalibacterium prausnitzii*, *Roseburia hominis*, *Eubacterium rectale*, *Anaerostipes* spp., and other members of the Firmicutes phylum [4,6,22]. Importantly, reduced abundance of these organisms may be accompanied by diminished representation of bacterial pathways involved in butyrate biosynthesis, indicating that the functional impairment of the microbiota extends beyond changes in microbial composition alone [4,24,25].

However, quantitative reductions in luminal SCFAs represent only one component of the metabolic defect. Experimental studies have demonstrated that epithelial cells exposed to the inflammatory environment of active UC may exhibit impaired uptake and intracellular utilization of butyrate, accompanied by alterations in monocarboxylate transporter expression, defective mitochondrial β-oxidation, and reduced oxidative metabolism [8,10,31]. Consequently, epithelial cells may undergo metabolic reprogramming characterized by increased glycolytic activity, mitochondrial dysfunction, oxidative stress, and diminished energy production. These findings support the concept that UC may involve both reduced microbial generation and impaired epithelial utilization of SCFAs, although the relative contribution of each mechanism may vary according to disease activity and experimental context [8,10,31].

Clinical observations further reinforce the potential biological importance of these metabolic abnormalities. Lower fecal butyrate concentrations have been associated in some studies with greater endoscopic or histological disease activity, elevated fecal calprotectin concentrations, impaired mucosal healing, and an increased likelihood of an unfavorable disease course [13,25,26,27,42]. Several longitudinal observations also suggest that restoration of SCFA-producing bacterial populations may accompany successful therapeutic responses, whereas persistent depletion of butyrate-producing taxa may be associated with sustained inflammatory activity and incomplete mucosal recovery. These associations, however, do not establish whether impaired SCFA production is a primary driver of inflammation or a consequence of the inflammatory intestinal environment [24,25].

Evidence from animal models provides additional mechanistic support. Experimental colitis induced by dextran sulfate sodium (DSS), trinitrobenzene sulfonic acid (TNBS), or other chemical models has been associated with reductions in colonic SCFA concentrations together with disruption of epithelial energy metabolism and barrier integrity [16,43,44,45]. Restoration of endogenous SCFA availability through dietary fiber supplementation, prebiotic administration, or microbiome-directed interventions may attenuate intestinal inflammation, improve epithelial regeneration, and restore aspects of mucosal homeostasis [13,14,15,35,38]. These experimental observations provide important mechanistic support for a causal role of microbial metabolism in intestinal inflammation, although their direct translation to human UC remains uncertain.

Nevertheless, SCFA deficiency should not be viewed as an isolated biochemical abnormality. Rather, it represents one component of a broader metabolic network involving bile acid metabolism, tryptophan-derived microbial metabolites, succinate accumulation, and altered microbial cross-feeding [10,20,22,23]. These interconnected pathways may collectively influence epithelial metabolic competence and immune homeostasis, underscoring the importance of evaluating microbial function as an integrated system rather than focusing exclusively on individual metabolites.

Taken together, current evidence supports the concept that impaired SCFA production and utilization constitute important features of UC pathophysiology and provide a biologically plausible target for therapeutic intervention. These observations provide a mechanistic rationale for strategies aimed at restoring endogenous microbial metabolism through dietary interventions, prebiotics, microbiome-directed therapies, and fermentable fibers capable of enhancing physiological SCFA production. This concept provides the biological basis for the subsequent discussion of pectin as a microbiome-modulating therapeutic candidate.

## 3. Therapeutic Strategies Targeting Microbial SCFA Restoration

The recognition of SCFA deficiency as an important feature of UC has stimulated growing interest in therapeutic strategies aimed at restoring microbial metabolic homeostasis. Unlike conventional anti-inflammatory therapies, which primarily suppress immune activation, SCFA-directed interventions seek to correct upstream metabolic abnormalities by increasing the availability or biological activity of beneficial microbial metabolites. These approaches include direct administration of SCFAs, enhancement of endogenous microbial SCFA production through fermentable dietary substrates, modulation of gut microbial ecology, and emerging microbiome-directed interventions. Although the biological rationale supporting these strategies is compelling, clinical translation has proved considerably more challenging. Therapeutic efficacy appears to depend on multiple factors, including disease activity, route of administration, regional drug delivery, microbial composition, dietary substrate availability, host metabolic responsiveness, and treatment duration. Consequently, current evidence supports the biological plausibility of SCFA-directed metabolic therapy but remains insufficient to justify routine clinical implementation. The following sections summarize the principal therapeutic approaches currently investigated to restore endogenous SCFA metabolism and evaluate their clinical relevance in patients with UC [8,32,39,41].

### 3.1. Direct SCFA Replacement

The recognition that reduced SCFA availability may contribute to the pathophysiology of UC prompted early attempts to restore colonic SCFA concentrations through direct administration. Because butyrate represents an important energy substrate for colonocytes and exerts multiple epithelial and immunomodulatory effects, local replacement therapy was initially regarded as a rational strategy for correcting epithelial metabolic insufficiency independently of changes in the intestinal microbiota.

The majority of early clinical studies investigated butyrate enemas, aiming to deliver high luminal concentrations directly to the inflamed distal colon [46,47,48,49]. Randomized and uncontrolled studies reported improvements in some patients with mild-to-moderate distal UC, including clinical symptoms, endoscopic findings, and histological inflammatory activity. These effects were attributed to restoration of colonocyte energy metabolism, enhancement of epithelial repair, modulation of tight-junction function, suppression of NF-κB signaling, and reduction in pro-inflammatory cytokine responses. Despite these encouraging findings, subsequent clinical trials produced inconsistent results [48]. Considerable heterogeneity existed with regard to butyrate concentration, formulation, treatment duration, disease activity, concomitant medication, and study design. Patient adherence also represented a practical limitation because rectal administration is inconvenient, particularly when prolonged treatment is required. Consequently, although butyrate enemas provided evidence of biological activity, they did not achieve widespread clinical adoption.

Attempts have also been made to administer SCFAs orally. However, conventional oral formulations face substantial pharmacokinetic challenges because SCFAs are efficiently absorbed in the proximal gastrointestinal tract before reaching the colon, where their therapeutic effects are primarily required [10,31]. Although colon-targeted delivery systems and microencapsulation technologies have been developed to overcome this limitation, clinical evidence remains limited, and no oral SCFA formulation has become part of routine UC management. An additional limitation of direct SCFA replacement is that it addresses the availability of a downstream microbial metabolite without necessarily correcting the underlying ecological and metabolic disturbances responsible for SCFA deficiency. Reduced abundance of butyrate-producing bacteria, disruption of microbial cross-feeding networks, altered fermentation capacity, and impaired epithelial responsiveness may persist despite exogenous SCFA administration. Consequently, the therapeutic effect of direct replacement may be limited unless the broader intestinal microbial ecosystem and host metabolic response are also addressed. Current evidence therefore suggests that direct SCFA replacement provides proof of biological principle rather than a definitive therapeutic solution [46,47,48,49].

Clinical studies support important anti-inflammatory and epithelial-protective properties of butyrate, but the modest and variable efficacy observed across clinical trials indicates that replacement of a single microbial metabolite may be insufficient to reverse the complex metabolic dysfunction characteristic of UC. These limitations have shifted scientific interest toward strategies that enhance endogenous microbial SCFA production, thereby restoring physiological host–microbiome metabolic interactions rather than replacing a single metabolite.

### 3.2. Strategies to Enhance Endogenous SCFA Production

Growing recognition of the limitations associated with direct SCFA replacement has shifted research toward therapeutic strategies designed to restore endogenous microbial SCFA production. Rather than supplying butyrate exogenously, these approaches seek to re-establish physiological metabolic interactions between the intestinal microbiota and the host by providing fermentable substrates that support beneficial microbial communities and their metabolic activity [8,20,22,39,41]. This concept represents a fundamental transition from metabolite replacement therapy toward restoration of microbial ecosystem function.

Among the various approaches investigated, dietary modulation has emerged as a physiological means of increasing colonic SCFA production. Diets enriched in complex, non-digestible carbohydrates provide fermentable substrates for anaerobic bacterial fermentation, resulting in increased production of acetate, propionate, and butyrate. The magnitude and composition of this response depend not only on the amount and type of dietary fiber but also on the composition and functional capacity of the resident microbiota, highlighting the importance of host–microbiome interactions in determining therapeutic effects [8,20,39,50,51].

Prebiotics constitute one of the best-studied dietary strategies for enhancing endogenous SCFA production. According to the current consensus definition, prebiotics are selectively utilized substrates that confer health benefits through modulation of the gut microbiota. Fermentable fibers such as inulin, fructooligosaccharides (FOSs), galactooligosaccharides (GOSs), resistant starch, arabinoxylans, β-glucans, and pectin can stimulate microbial taxa capable of SCFA production, including members of the Faecalibacterium, Roseburia, Anaerostipes, and Eubacterium genera [8,31,39,43]. Enhanced fermentation of these substrates may increase luminal SCFA availability and modify microbial metabolic activity. Importantly, restoration of endogenous SCFA production does not necessarily depend on expansion of a single bacterial species but on preservation of microbial cross-feeding networks [9,20,21]. Primary degraders hydrolyze complex polysaccharides into intermediate metabolites that can subsequently be utilized by secondary fermenters to generate butyrate and other SCFAs. Disruption of these cooperative microbial interactions is increasingly recognized as an important component of functional dysbiosis in UC. Consequently, interventions capable of restoring metabolic networks may produce broader biological effects than direct administration of individual metabolites [20,22,23].

Beyond increasing SCFA availability, fermentable fibers may exert additional biological effects that support intestinal homeostasis. Fermentation can modify luminal pH, influence microbial ecological composition, affect bile acid transformation, and alter tryptophan-derived microbial signaling pathways, including pathways involving the aryl hydrocarbon receptor (AhR) [20,22,28]. These mechanisms may contribute to epithelial barrier function and immune regulation. However, the extent to which these effects translate into clinically meaningful improvements in human UC remains incompletely established and should be distinguished from evidence derived from experimental models.

Nevertheless, therapeutic responses to dietary fiber are not uniform. Considerable inter-individual variability may arise from differences in baseline microbiota composition, disease activity, dietary habits, antibiotic exposure, and other host-related factors [24,25,26,27,29]. In patients with marked dysbiosis, limited availability or functional impairment of SCFA-producing microorganisms may reduce the capacity to ferment particular dietary substrates, thereby attenuating their metabolic effects. These observations have contributed to increasing interest in personalized microbiome-directed nutritional interventions based on individual microbial and metabolic profiles.

Among currently available fermentable fibers, pectin possesses several characteristics that make it an attractive candidate for further investigation. Owing to its complex polysaccharide structure and high fermentability, pectin can serve as a substrate for specific microbial populations and may promote SCFA generation through microbial fermentation [45,52,53,54]. In addition to its potential effects on SCFA production, pectin may influence epithelial barrier function, immune regulation, oxidative stress, and microbial ecological stability through complementary mechanisms. These properties provide the biological rationale for its detailed evaluation in the following section, while the extent of its clinical efficacy in UC remains an important question for future investigation.

**Figure 3:**
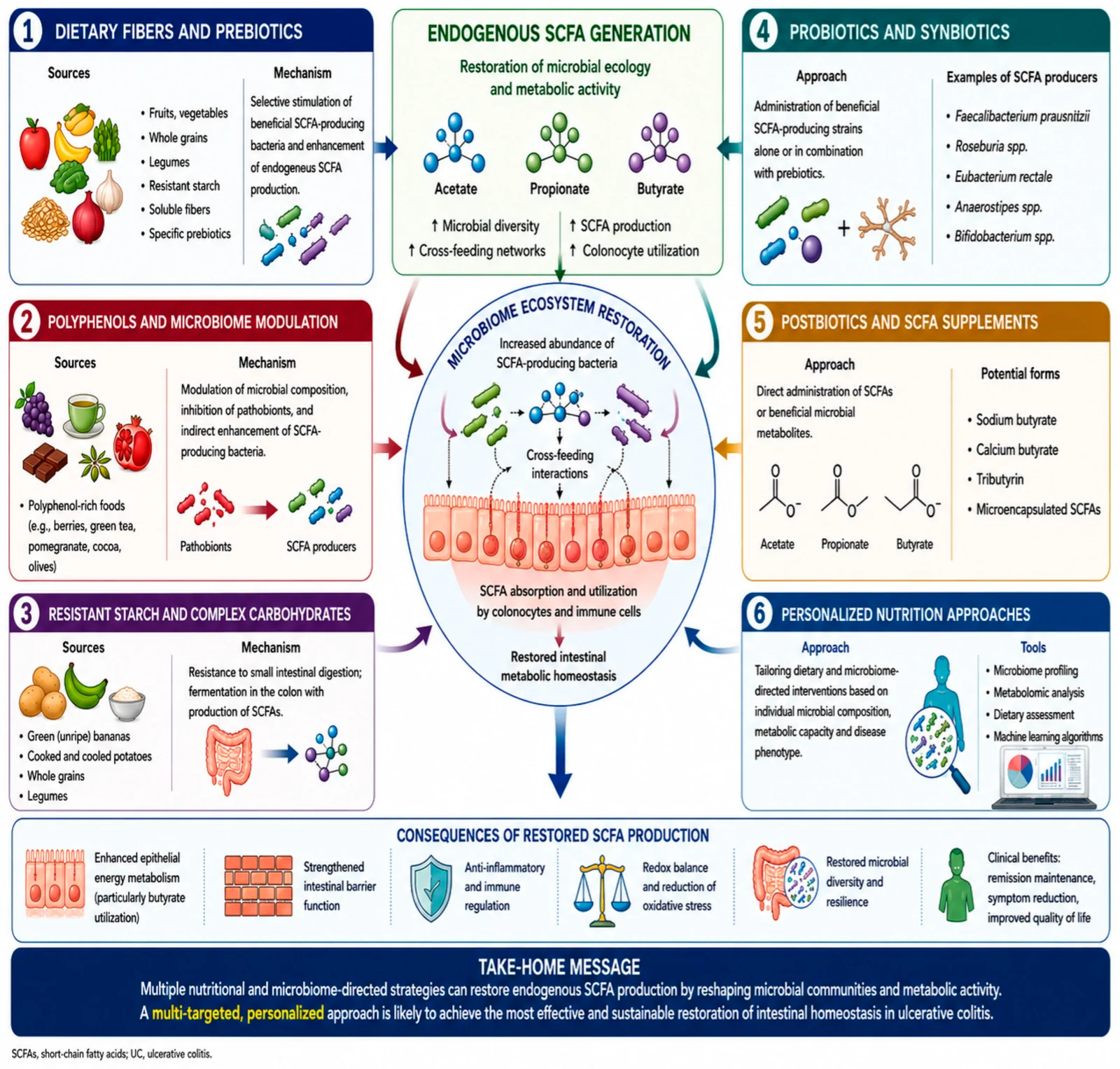
Strategies for restoring microbial SCFA production in UC: from nutritional interventions to endogenous SCFA generation. Multiple complementary nutritional and microbiome-directed strategies have been investigated to restore endogenous SCFA production in UC by promoting recovery of microbial metabolic activity rather than direct replacement of microbial metabolites. Dietary fibers and prebiotics selectively stimulate SCFA-producing microorganisms, whereas resistant starch provides fermentable substrates that enhance microbial fermentation and butyrate synthesis. Polyphenol-rich foods indirectly support SCFA production by modulating microbial community composition, suppressing pathobionts, and enriching beneficial commensal bacteria. Probiotics and synbiotics further contribute by introducing or supporting microorganisms capable of participating in SCFA-generating metabolic networks, while postbiotics and exogenous SCFA formulations may provide direct supplementation of microbial metabolites when endogenous production is impaired. Although these approaches differ in their primary mechanisms of action, they converge toward restoration of microbial ecosystem function through increased abundance of SCFA-producing bacteria; re-establishment of microbial cross-feeding interactions; enhanced endogenous production of acetate, propionate, and butyrate; and improved utilization of these metabolites by colonocytes. Recovery of microbial metabolic competence promotes restoration of epithelial bioenergetics, reinforcement of intestinal barrier integrity, attenuation of inflammatory signaling, reduction in oxidative stress, stabilization of microbial ecological resilience, and maintenance of intestinal homeostasis. The final panel illustrates the emerging concept of personalized microbiome-directed nutrition, in which dietary interventions are tailored according to individual microbial composition, metabolic capacity, disease phenotype, and multi-omics biomarkers. This precision medicine approach recognizes that therapeutic efficacy depends not only on the administered nutritional substrate but also on the functional capacity of the intestinal microbiome to ferment complex carbohydrates and generate biologically active SCFAs. Consequently, restoration of endogenous microbial metabolism represents the principal therapeutic objective, while direct metabolite supplementation should be regarded as a complementary strategy for selected clinical settings. Overall, the figure emphasizes that restoration of microbial SCFA production is a systems-level therapeutic strategy integrating nutritional intervention, microbial ecology, and host metabolism. Within this framework, pectin emerges as one of the most biologically plausible microbiome-directed metabolic interventions because of its capacity to selectively stimulate pectinolytic microorganisms, reinforce microbial cross-feeding networks, and enhance sustained endogenous butyrate production, thereby providing the mechanistic basis for the subsequent sections of this review.

Figure 3 outlines strategies for restoring microbial SCFA production in UC patients.

The characteristics that define an ideal SCFA-restoring prebiotic are summarized in Table 3. The therapeutic objective is not simply to increase luminal SCFA concentrations but to restore the functional metabolic capacity of the intestinal microbiome, thereby re-establishing physiological host–microbiome interactions that sustain epithelial homeostasis and immune regulation. Among the currently available fermentable fibers, pectin fulfills many of these criteria and is therefore discussed in detail in the following section.

## 4. Pectin

### 4.1. Structure, Classification, and Physicochemical Properties

Pectin is a heterogeneous family of complex plant-derived polysaccharides that constitutes a major structural component of the primary cell wall and middle lamella of higher plants. Owing to its distinctive molecular architecture, physicochemical properties, and high fermentability, pectin has attracted increasing interest as a microbiome-directed dietary substrate capable of modifying microbial metabolism and supporting intestinal homeostasis. Unlike many conventional dietary fibers, pectin exhibits substantial structural diversity, and this heterogeneity may influence its solubility, viscosity, microbial accessibility, fermentation kinetics, and ultimately its biological effects within the gastrointestinal tract.

The pectin molecule consists of several structurally distinct domains. Homogalacturonan (HG), which represents the major structural domain in most pectins, comprises linear chains of α-(1→4)-linked D-galacturonic acid residues that may undergo variable degrees of methyl esterification and acetylation. Interspersed with or associated with HG are the more structurally complex rhamnogalacturonan-I (RG-I) and rhamnogalacturonan-II (RG-II) domains [45,54]. RG-I contains alternating rhamnose and galacturonic acid residues with branched arabinan- and galactan-rich side chains, whereas RG-II possesses a highly complex and conserved structure containing several rare monosaccharides. The relative contribution and organization of these domains vary according to botanical source and extraction and processing procedures. Such structural characteristics influence the susceptibility of pectin to microbial degradation after its passage through the upper gastrointestinal tract [52,55].

One of the most important physicochemical characteristics of pectin is its degree of methyl esterification (DM), which contributes substantially to its functional behavior. Pectins are conventionally classified as high-methoxyl (HM) pectins when more than 50% of the galacturonic acid residues are methyl-esterified and as low-methoxyl (LM) pectins when the degree of esterification is below 50%. HM pectins can form gels under acidic conditions in the presence of high concentrations of soluble solids, whereas LM pectins undergo calcium-mediated gelation through the so-called “egg-box” mechanism. Although these properties are particularly relevant to the food and pharmaceutical applications of pectin, the degree of esterification may also influence microbial accessibility, fermentation behavior, and the biological availability of pectin-derived microbial metabolites.

The biological behavior of pectin is further influenced by molecular weight, degree and pattern of branching, acetylation, neutral sugar composition, HG/RG-I ratio, botanical origin, and extraction and processing conditions. Commercial pectins are commonly obtained from citrus peels and apple pomace, although pectins derived from sugar beet, sunflower, and other plant sources may exhibit substantially different structural characteristics. These differences can affect hydration, viscosity, water-holding capacity, susceptibility to microbial degradation, fermentation kinetics, and the pattern of microbial metabolites generated during colonic fermentation. Consequently, pectin should not be regarded as a single uniform compound but rather as a structurally diverse family of polysaccharides whose biological properties depend on molecular architecture and source [45,52,53,54].

Unlike readily digestible carbohydrates, pectin largely escapes enzymatic degradation in the upper gastrointestinal tract and reaches the colon, where it becomes available for microbial utilization [53]. Its fermentation depends on the presence and functional capacity of pectinolytic microorganisms and involves complex metabolic interactions among different microbial populations. Pectin-derived fermentation products include acetate, propionate, and butyrate, with the final metabolite profile depending on both the structural characteristics of the substrate and the composition and metabolic capacity of the resident microbiota [53,54,55]. Thus, the physicochemical properties of pectin are directly linked to its potential role as a substrate for restoration of endogenous microbial SCFA production.

Importantly, the biological effects of pectin may extend beyond its role as a fermentable carbohydrate. Its physicochemical characteristics can influence luminal viscosity and hydration, interactions with bile acids and the mucus layer, and the spatial organization of microbial communities within the intestinal lumen. These properties provide a potential mechanistic link between the molecular structure of pectin and its effects on the intestinal ecosystem. However, such effects are likely to be highly dependent on pectin structure, dose, botanical source, processing, and the metabolic characteristics of the individual microbiota.

Accordingly, the current literature supports an important structure–function relationship in pectin biology: different pectins should not be assumed to be biologically equivalent. Differences in degree of esterification, molecular weight, HG/RG-I composition, side-chain architecture, botanical source, and extraction method may substantially influence their fermentability and biological activity. This heterogeneity is particularly relevant when considering pectin as a potential therapeutic substrate in ulcerative colitis, because the biological effects observed with one pectin preparation cannot necessarily be extrapolated to all pectin preparations [45,52,53,54].

Within this framework, pectin can be considered from two complementary perspectives. First, bioactive pectin represents the therapeutic substrate itself, acting through interactions with the intestinal microbiota, microbial metabolism, epithelial physiology, and mucosal immune pathways. Second, pectin-based delivery systems represent a pharmaceutical and pharmacotechnical application in which the physicochemical properties of pectin are exploited to achieve controlled, bioadhesive, pH-responsive, enzyme-responsive, or colon-targeted delivery of other therapeutic agents. These two fields are mechanistically related but conceptually distinct and should therefore be considered separately when evaluating the therapeutic potential of pectin in UC.

From a therapeutic perspective, the biological effects attributed to pectin can be broadly organized into several interconnected domains, including modulation of intestinal inflammation, reinforcement of epithelial barrier function, alteration of gut microbial ecology and SCFA production, immunomodulation, and exploitation of pectin as a biomaterial for targeted drug delivery. The evidence supporting these effects, however, varies substantially according to the pectin preparation, experimental model, and biological endpoint examined. Therefore, these mechanisms should not be interpreted as established clinical effects in UC but rather as components of a mechanistic framework that requires validation in appropriately designed translational and clinical studies.

In conclusion, the structural complexity and physicochemical versatility of pectin provide the mechanistic foundation for its multiple biological effects within the intestinal ecosystem. Rather than acting solely as a source of fermentable carbohydrate, pectin may function as a microbiome-directed biomaterial capable of influencing microbial ecology, metabolic activity, epithelial physiology, and mucosal immune homeostasis. These structure-dependent properties provide the basis for examining, in the following sections, the microbiological and metabolic mechanisms through which pectin may enhance endogenous SCFA production and potentially contribute to maintenance of remission in UC.

**Figure 4:**
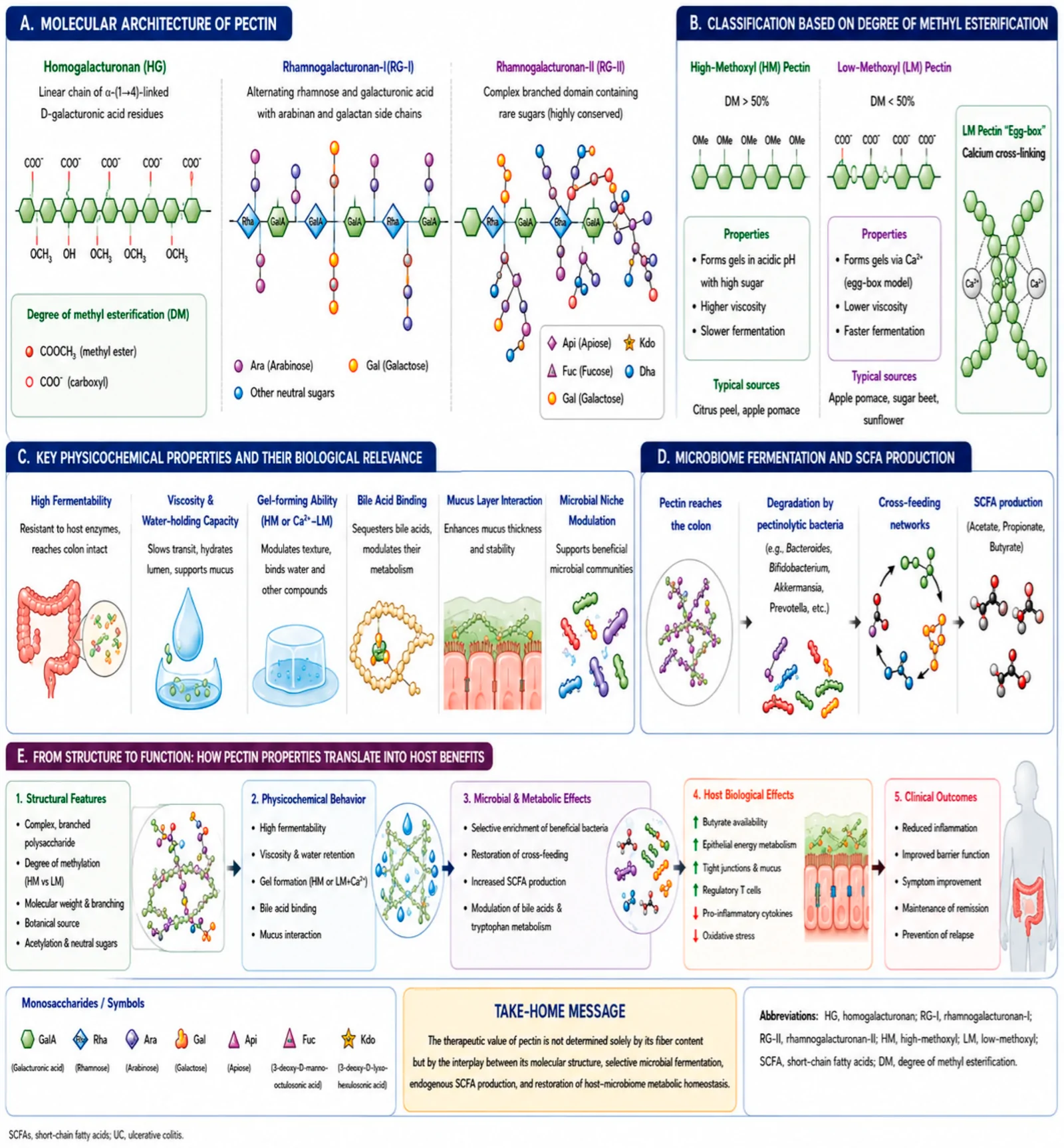
Structural organization of pectin and determinants of its biological activity in the colon. *Pectin* is a structurally heterogeneous plant-derived polysaccharide whose biological activity is determined by its molecular architecture and physicochemical properties. The molecule consists primarily of homogalacturonan (HG), rhamnogalacturonan-I (RG-I), and rhamnogalacturonan-II (RG-II) domains, while its degree of methyl esterification (high-methoxyl versus low-methoxyl pectin), molecular weight, branching pattern, acetylation, and botanical origin influence its functional characteristics. Because pectin escapes digestion in the upper gastrointestinal tract, it reaches the colon intact, where it undergoes selective microbial fermentation by pectinolytic bacteria. This process promotes microbial cross-feeding networks and enhances endogenous production of SCFAs, particularly butyrate. In addition to serving as a fermentable substrate, pectin contributes to intestinal homeostasis by modulating luminal viscosity, water retention, bile acid metabolism, mucus layer stability, and microbial ecological organization. Collectively, these structural and physicochemical characteristics translate into enhanced epithelial energy metabolism, improved barrier integrity, reduced inflammatory signaling, and maintenance of mucosal homeostasis, providing the mechanistic basis for the therapeutic potential of pectin in UC [16,52,53,55,56].

Figure 4 illustrates the structural organization of pectin and the principal determinants of its biological activity within the colon.

### 4.2. Microbial Fermentation of Pectin and SCFA Production

Unlike digestible carbohydrates, pectin largely resists enzymatic degradation in the upper gastrointestinal tract and reaches the colon, where it becomes available for microbial fermentation [55]. This property represents a principal mechanism through which pectin may influence microbial ecology, metabolic activity, and host physiology. Rather than functioning solely as a source of dietary fiber, pectin can be considered a microbiome-directed substrate whose fermentation has the potential to enhance endogenous microbial metabolite production and modify host–microbiome metabolic interactions.

Degradation of pectin requires a coordinated enzymatic repertoire that includes pectin methylesterases, polygalacturonases, pectate lyases, rhamnogalacturonan hydrolases, and accessory glycosidases capable of acting on the structurally diverse homogalacturonan (HG), rhamnogalacturonan-I (RG-I), and rhamnogalacturonan-II (RG-II) domains. These enzymes are produced by specialized pectinolytic microorganisms, including members of the Bacteroides and Prevotella genera and selected Firmicutes. Initial degradation generates smaller oligosaccharides and monosaccharides that can subsequently enter interconnected microbial fermentation pathways. Importantly, pectin fermentation does not represent an isolated metabolic process but may involve extensive microbial cross-feeding, whereby metabolites generated by primary degraders become substrates for secondary fermenters. Through these cooperative interactions, fermentation intermediates can contribute to the generation of acetate, propionate, and butyrate, including butyrate production by organisms such as *Faecalibacterium prausnitzii*, *Roseburia* spp., *Anaerostipes* spp., and *Eubacterium* spp. The resulting metabolic network provides a mechanistic basis for linking pectin fermentation with restoration of microbial functions that are frequently impaired in UC [52,53,54,56].

The importance of this process extends beyond the abundance of individual bacterial taxa. The metabolic outcome of pectin fermentation depends on the structural characteristics of the substrate, the enzymatic capacity of the resident microbiota, and the availability of complementary metabolic pathways within the microbial community. Accordingly, pectin may support a functionally integrated microbial ecosystem rather than simply promoting expansion of a single bacterial population. Restoration of microbial cross-feeding and fermentation capacity may be particularly relevant in UC, in which disruption of microbial metabolic networks can coexist with substantial inter-individual variation in microbial composition. Nevertheless, the extent to which pectin consistently restores these networks in patients with UC remains incompletely defined.

The principal metabolic consequence of pectin fermentation is the generation of SCFAs, although the quantity and relative proportions of individual SCFAs vary according to pectin source, molecular structure, dose, fermentation conditions, and microbial composition. Increased availability of butyrate may support colonocyte oxidative metabolism and epithelial barrier function, whereas acetate and propionate participate in additional epithelial and systemic metabolic pathways [8,10,53]. Fermentation-associated changes in luminal pH may also influence microbial ecological conditions and the relative competitiveness of different bacterial populations. In addition, pectin fermentation may interact with other microbial metabolic pathways, including bile acid transformation and tryptophan-derived metabolite production, thereby extending its potential biological effects beyond SCFA generation alone [20,22,23,41].

Experimental studies indicate that pectin supplementation can increase intestinal or fecal concentrations of SCFAs and can modify the abundance or activity of SCFA-associated microbial populations. In experimental models of intestinal inflammation, these metabolic changes have been accompanied by improvements in epithelial barrier function, preservation of goblet-cell populations and mucus production, attenuation of inflammatory signaling, and reduction in histological injury. However, the magnitude and direction of these effects vary according to the pectin preparation, experimental model, dose, duration of administration, and baseline microbial ecosystem [16,44,45,52,53,54,55,56,57,58,59,60]. These observations therefore provide mechanistic and preclinical support for the potential of pectin to modify microbial metabolism, but they should not be interpreted as evidence of established therapeutic efficacy in human UC.

An emerging concept is that pectin may function as a systems-level metabolic modulator rather than simply as a conventional prebiotic. By simultaneously providing fermentable substrate, influencing microbial ecological interactions, modifying metabolite production, and potentially affecting epithelial and immune pathways, pectin may act at several interconnected levels of the host–microbiome metabolic network. This systems-level perspective provides a broader mechanistic framework for understanding the biological effects of pectin than SCFA generation alone. However, whether these interconnected effects translate into clinically meaningful improvement or sustained maintenance of remission in UC remains an important unanswered question requiring controlled human studies.

**Figure 5:**
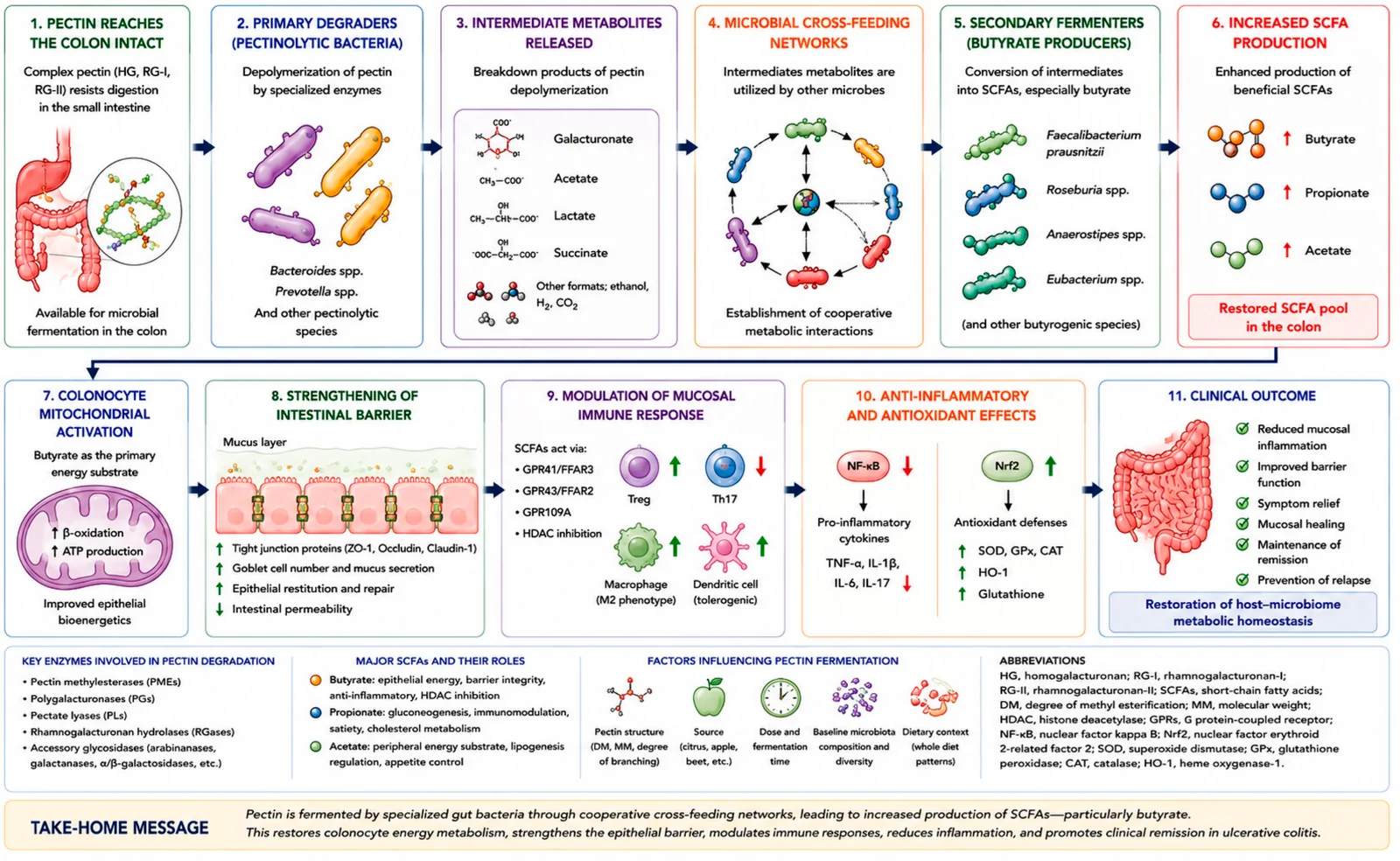
Microbial fermentation of pectin and restoration of host–microbiome metabolic homeostasis in UC. Pectin reaches the colon largely undigested, where it becomes available for microbial fermentation by pectinolytic microorganisms, including members of the genera Bacteroides and Prevotella. Initial degradation of the homogalacturonan (HG), rhamnogalacturonan-I (RG-I), and rhamnogalacturonan-II (RG-II) domains generates oligosaccharides and other fermentation intermediates that participate in interconnected microbial metabolic pathways. Through microbial cross-feeding, metabolites generated by primary degraders may subsequently support secondary fermenters, including butyrate-producing organisms such as *Faecalibacterium prausnitzii*, *Roseburia* spp., *Anaerostipes* spp., and *Eubacterium* spp. [20,21,28,33]. This process may increase endogenous production of SCFAs, particularly butyrate, thereby supporting colonocyte oxidative metabolism and epithelial barrier function. SCFAs may additionally act through G protein-coupled receptors, including FFAR2/GPR43, FFAR3/GPR41, and HCAR2/GPR109A, and through intracellular pathways including histone deacetylase (HDAC) inhibition, thereby influencing epithelial and immune responses. Beyond SCFA production, pectin fermentation may modify luminal pH, microbial ecological interactions, bile acid transformation, and tryptophan-derived microbial metabolism. Collectively, these mechanisms provide a proposed pathway through which pectin may influence epithelial integrity, mucosal immune regulation, and intestinal metabolic homeostasis [52,53,58]. The final clinical consequences, including improvement of disease activity, maintenance of remission, and prevention of relapse, remain to be established in appropriately designed human studies.

Figure 5 summarizes the proposed microbial and metabolic pathway through which pectin fermentation may contribute to restoration of host–microbiome metabolic homeostasis in UC.

### 4.3. Biological Effects of Pectin in Experimental Colitis

Experimental models of colitis have provided substantial preclinical evidence that pectin exerts broad protective effects against intestinal inflammation through coordinated modulation of microbial metabolism, epithelial function, oxidative stress, and mucosal immunity. Although differences exist among experimental protocols, animal species, pectin formulations, and disease models, a broadly consistent protective pattern has been observed, supporting the concept that pectin may act as a multifunctional microbiome-directed therapeutic substrate.

Most available preclinical evidence comes from chemically induced models of colitis, particularly dextran sulfate sodium (DSS)- and trinitrobenzene sulfonic acid (TNBS)-induced colitis. In these models, dietary pectin has generally been associated with attenuation of clinical disease severity, including body-weight loss, diarrhea, rectal bleeding, and disease activity scores. Histological examination has demonstrated reduced epithelial injury and inflammatory cell infiltration, preservation of crypt architecture, and improved mucosal integrity compared with untreated controls. An important component of the protective response appears to involve restoration of epithelial barrier function. Experimental studies have reported increased expression of tight-junction proteins, including zonula occludens-1 (ZO-1), occludin, and claudin-1, together with preservation of goblet-cell populations and mucin production. Maintenance of the mucus layer may further limit epithelial exposure to luminal microbial products and reduce intestinal permeability. Collectively, these findings indicate that improvement of epithelial barrier integrity represents an important component of the protective effects attributed to pectin in experimental colitis [16,44,45,52,53,54,55,56,57,58,59,60].

Pectin also exerts important effects on microbial metabolism. In experimental studies, pectin supplementation has been associated with increased abundance of SCFA-producing bacterial taxa and with increased colonic concentrations of acetate, propionate, and, in some models, butyrate. Enhanced endogenous SCFA availability may support colonocyte mitochondrial β-oxidation, energy production, and epithelial repair. Importantly, these metabolic effects may involve interactions among different microbial populations and restoration of microbial cross-feeding pathways, rather than expansion of a single bacterial taxon [16,44,52,53]. Thus, pectin may influence the functional capacity of the intestinal microbial ecosystem in addition to modifying its taxonomic composition. However, the extent to which these effects occur consistently across different pectin preparations and translate into clinically relevant metabolic changes in patients with UC remains uncertain [16,44,45,56].

In addition to its metabolic actions, pectin markedly suppresses mucosal inflammation. Across multiple experimental models, treatment is associated with reduced tissue expression of TNF-α, IL-1β, IL-6, and IL-17, accompanied by increased production of anti-inflammatory mediators such as IL-10. Several studies further demonstrate inhibition of NF-κB activation, attenuation of NLRP3 inflammasome overactivation, and increased differentiation of regulatory T cells, collectively promoting restoration of immune homeostasis [57,58,59,60,61].

Oxidative stress represents another important therapeutic target influenced by pectin. Experimental colitis is characterized by excessive production of reactive oxygen species (ROS), mitochondrial dysfunction, and depletion of endogenous antioxidant defenses. Pectin supplementation has been associated with attenuation of oxidative injury, including reduced lipid peroxidation and restoration of antioxidant defenses such as superoxide dismutase (SOD), catalase (CAT), and glutathione peroxidase (GPx). In several experimental models, these effects have been accompanied by increased expression or activation of antioxidant pathways, including nuclear factor erythroid 2-related factor 2 (Nrf2) and heme oxygenase-1 (HO-1). These observations suggest that the antioxidant effects of pectin may contribute to preservation of epithelial integrity and attenuation of mucosal injury [52,59,61]. However, the available evidence is predominantly derived from experimental models, and the relative contribution of direct antioxidant activity versus indirect effects mediated through microbiota-derived metabolites and improved epithelial metabolic function remains incompletely defined [16,54,60].

The biological effects of pectin are influenced by its structural characteristics. Differences in molecular weight, degree of methyl esterification, branching pattern, and botanical source affect fermentation kinetics, microbial selectivity, SCFA production, and immunomodulatory activity. Low-methoxyl and high-methoxyl pectins may therefore exhibit distinct biological profiles, although direct comparative studies remain limited [45,52,53,54]. These observations suggest that optimization of pectin formulation may further enhance its therapeutic potential [16,52,55,56].

In summary, experimental studies consistently demonstrate that pectin acts simultaneously on multiple interconnected pathogenic pathways in ulcerative colitis. Rather than functioning solely as a fermentable dietary fiber, pectin restores microbial metabolic competence, reinforces epithelial barrier function, attenuates oxidative stress, modulates innate and adaptive immune responses, and promotes mucosal healing. The convergence of these complementary mechanisms provides strong biological support for the translational evaluation of pectin as an adjunctive microbiome-directed metabolic therapy in UC. However, despite all this progress, most of the studies continue to be preclinical with few studies performed in humans [12,17] (including an FMT-plus-pectin study and a negative ileal pouch study).

Table 4 shows the principal biological effects of pectin in experimental models of UC.

### 4.4. Mechanistic Basis of Pectin-Mediated Protection

The beneficial effects of pectin in UC cannot be attributed to a single biological pathway. Rather, available experimental evidence indicates that pectin may exert multifaceted and interconnected effects involving microbial metabolism, epithelial bioenergetics, immune regulation, oxidative stress, and intestinal barrier maintenance. These mechanisms may operate synergistically to restore host–microbiome metabolic homeostasis rather than simply suppressing intestinal inflammation. A central component of the proposed mechanism is the enhancement of endogenous SCFA production through microbial fermentation. Increased availability of microbial SCFAs, particularly butyrate, may support colonocyte mitochondrial β-oxidation, ATP generation, and physiological oxidative metabolism, thereby contributing to epithelial differentiation, proliferation, and mucosal repair. In parallel, SCFAs generated during pectin fermentation can activate signaling pathways through both receptor-dependent and receptor-independent mechanisms. Extracellular signaling involves the G protein-coupled receptors FFAR2 (GPR43), FFAR3 (GPR41), and HCAR2 (GPR109A), which participate in the regulation of epithelial function, leukocyte responses, cytokine production, and innate immune activity. Intracellularly, butyrate can inhibit histone deacetylases (HDACs), thereby modifying transcriptional programs involved in epithelial differentiation, immune regulation, and inflammatory responses [8,9,52,53,57,58]. Importantly, these mechanisms are well established for SCFAs, whereas their specific contribution to the biological effects of pectin remains primarily supported by experimental studies and should therefore be considered mechanistically plausible rather than definitively established in human UC.

These complementary signaling mechanisms converge on several key inflammatory pathways. Experimental studies indicate that pectin supplementation can attenuate activation of the NF-κB pathway, with consequent reductions in the expression of pro-inflammatory mediators such as TNF-α, IL-1β, IL-6, and IL-17, although the magnitude and reproducibility of these effects vary according to the experimental model, pectin source, and formulation. In parallel, pectin-associated modulation of the intestinal microbiota and its metabolites may favor anti-inflammatory pathways, including increased IL-10 production and enhancement of regulatory T-cell responses. Changes in macrophage phenotype, particularly a shift toward a more reparative or anti-inflammatory profile, have also been reported in experimental models [8,9,11,12]. Collectively, these findings suggest that the immunomodulatory effects of pectin are not attributable to direct inhibition of a single inflammatory mediator but may result from coordinated interactions between microbial metabolites, epithelial signaling, and innate and adaptive immune pathways. Importantly, however, most of the available evidence derives from preclinical studies, and the extent to which these mechanisms operate in patients with UC remains to be established [57,58,60,61].

Pectin also exerts important cytoprotective actions through modulation of oxidative stress. In experimental models of intestinal inflammation, pectin supplementation has been associated with attenuation of oxidative injury and enhancement of endogenous antioxidant defenses, including increased activities or expression of superoxide dismutase, catalase, glutathione peroxidase, and heme oxygenase-1. These effects may be related, at least in part, to modulation of the Nrf2-dependent antioxidant response and to the broader metabolic consequences of enhanced microbial fermentation. By limiting oxidative damage to epithelial cells and preserving mitochondrial function, these mechanisms may contribute to maintenance of epithelial viability and mucosal repair during intestinal inflammation. However, the available evidence for Nrf2 activation and specific antioxidant pathways is derived predominantly from experimental models, and their relative contribution to the effects of pectin in human UC remains uncertain [52,59,61].

Maintenance of epithelial barrier integrity represents another potentially important mechanism of pectin-mediated protection. Experimental studies indicate that pectin supplementation may increase the expression of tight-junction proteins, including zonula occludens-1, occludin, and claudins, while also promoting goblet-cell preservation, mucin production, and maintenance of the mucus layer. These effects may be mediated both directly by interactions between pectin and the intestinal mucosal environment and indirectly through enhanced microbial fermentation and SCFA production. Improved barrier function may consequently reduce epithelial permeability and exposure of the mucosal immune system to luminal microbial products [8,17,39,44,45,57,58]. Nevertheless, the extent to which these observations are directly attributable to pectin itself, rather than to secondary changes in the microbiota and SCFA availability, remains incompletely defined. Thus, barrier reinforcement should be considered an important component of the proposed mechanism of action of pectin, but not yet an independently established therapeutic effect in patients with UC.

Beyond these established pathways, pectin may also influence additional components of the host–microbiome metabolic network, although the evidence supporting these effects is less extensive and is derived predominantly from experimental and mechanistic studies. Pectin fermentation can modify microbial bile acid transformation and thereby potentially influence downstream signaling through the farnesoid X receptor (FXR) and G protein-coupled bile acid receptor 1 (TGR5). Similarly, changes in microbial metabolism of tryptophan may alter the availability of aryl hydrocarbon receptor (AhR) ligands, with potential consequences for epithelial integrity and mucosal immune regulation. Pectin may also contribute to restoration of microbial ecological stability by supporting cooperative cross-feeding interactions among metabolically complementary bacterial populations [20,22,23,41,53,54]. These effects are biologically plausible and may contribute to the broader consequences of pectin supplementation; however, their specific contribution to protection against colitis remains incompletely defined. Accordingly, these pathways should currently be regarded as complementary and emerging components of the proposed mechanism rather than as established primary mechanisms of pectin-mediated protection in UC.

However, these biological actions should not be regarded as independent events but as components of an integrated therapeutic cascade. Restoration of microbial fermentation increases SCFA production, which improves epithelial bioenergetics, reinforces barrier integrity, attenuates inflammatory signaling, reduces oxidative stress, and promotes mucosal healing. Positive feedback between these mechanisms further stabilizes microbial ecology and supports long-term maintenance of intestinal homeostasis. This systems-based model distinguishes pectin from conventional pharmacological therapies that target individual inflammatory mediators.

**Figure 6:**
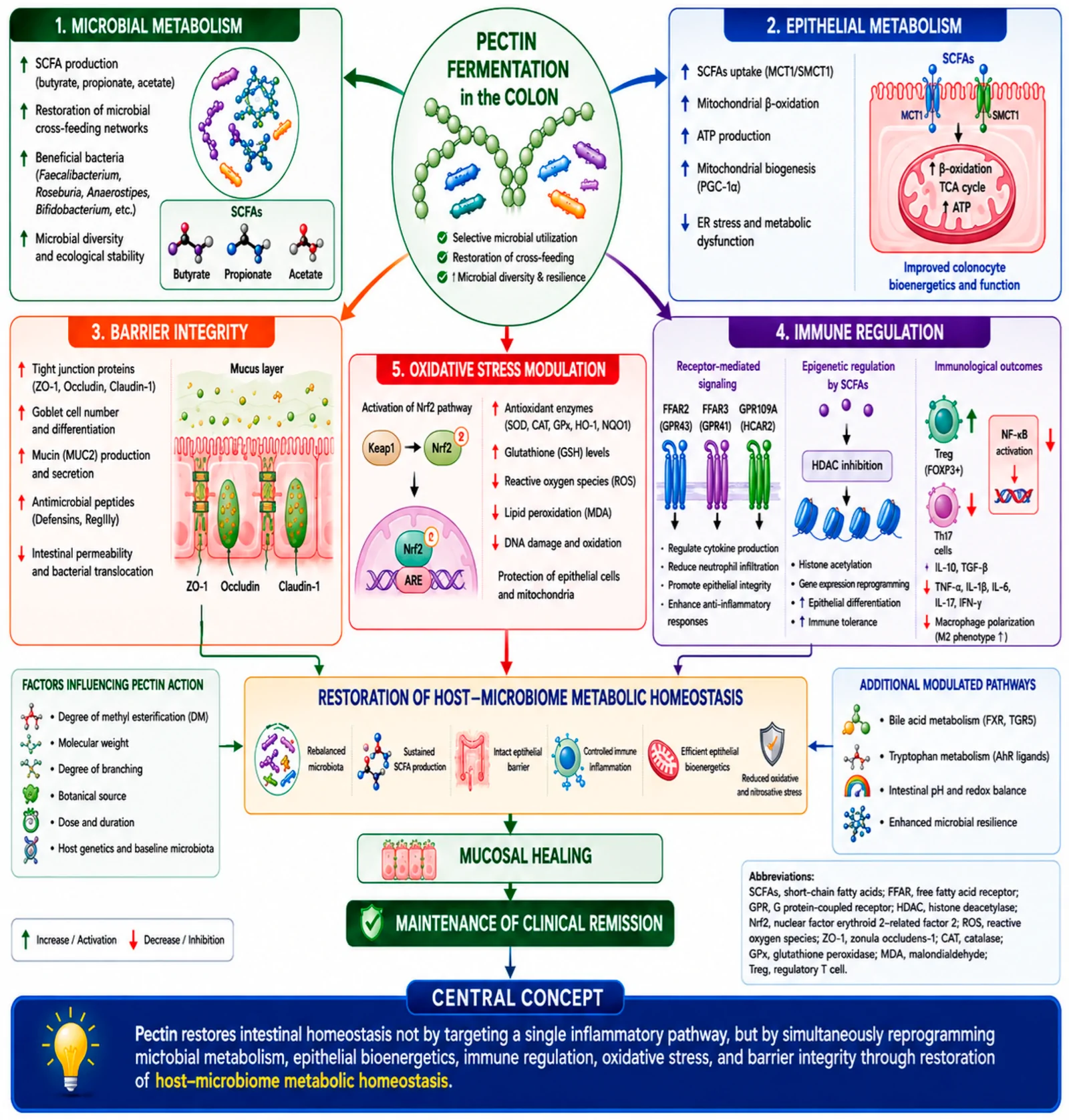
Integrated molecular mechanisms underlying pectin-mediated protection in UC. Pectin functions as a microbiome-directed metabolic regulator that restores host–microbiome metabolic homeostasis through multiple interconnected biological pathways. Following resistance to digestion in the upper gastrointestinal tract, pectin undergoes selective fermentation by pectinolytic microorganisms within the colon, promoting reconstruction of microbial cross-feeding networks and increased endogenous production of SCFAs, particularly butyrate. Restoration of SCFA availability enhances colonocyte mitochondrial β-oxidation and oxidative phosphorylation, improving ATP generation and epithelial bioenergetics while reducing metabolic stress. SCFAs simultaneously activate G protein-coupled receptors (FFAR2/GPR43, FFAR3/GPR41, and HCAR2/GPR109A) and inhibit histone deacetylases (HDACs), resulting in coordinated regulation of inflammatory gene expression, expansion of regulatory T cells, suppression of NF-κB signaling, and reduced production of pro-inflammatory cytokines. In parallel, pectin-mediated restoration of microbial metabolism reinforces epithelial barrier function by increasing expression of tight-junction proteins (ZO-1, occludin, and claudins), stimulating goblet-cell differentiation, enhancing mucin secretion, and reducing intestinal permeability. Activation of the Nrf2 antioxidant pathway further protects epithelial cells from oxidative injury by increasing endogenous antioxidant enzymes, including superoxide dismutase (SOD), catalase (CAT), glutathione peroxidase (GPx), and heme oxygenase-1 (HO-1). Additional systems-level effects include modulation of bile acid metabolism, regulation of tryptophan-derived aryl hydrocarbon receptor (AhR) ligands, stabilization of microbial ecological networks, and restoration of functional metabolic interactions between the intestinal microbiota and the host [16,45,54,56,57,59]. These complementary mechanisms converge to promote epithelial repair, mucosal healing, attenuation of chronic intestinal inflammation, and long-term maintenance of clinical remission. Rather than acting as a simple fermentable dietary fiber, pectin functions as a systems-level microbiome-directed metabolic therapy capable of simultaneously targeting microbial ecology, epithelial metabolism, immune regulation, oxidative stress, and barrier integrity [20,21,28,33], thereby providing a comprehensive biological framework for its potential role as an adjunctive therapeutic strategy in UC.

Figure 6 shows the integrated molecular mechanisms underlying pectin-mediated protection in UC.

### 4.5. Clinical Evidence and Translational Potential

Despite the compelling mechanistic rationale and consistently favorable findings obtained in experimental models, clinical evidence supporting the use of pectin in UC remains limited and heterogeneous. To date, only a small number of human studies have evaluated pectin supplementation, either as a dietary intervention or as a component of combined microbiome-directed nutritional strategies [17]. Accordingly, translation of the encouraging preclinical findings into routine clinical practice remains an important unmet research priority. The available human evidence suggests that pectin is generally well tolerated and may influence intestinal microbial composition and metabolic activity; however, the magnitude and clinical relevance of these effects remain uncertain. In selected exploratory studies, pectin-containing interventions have been associated with changes in fecal short-chain fatty acid concentrations, modulation of SCFA-producing bacterial taxa, and alterations in markers of intestinal inflammation or barrier function [17]. Nevertheless, these observations derive predominantly from small studies, heterogeneous interventions, or combined treatment approaches, and therefore cannot establish an independent therapeutic effect of pectin in UC. Importantly, direct evidence demonstrating that pectin supplementation produces sustained clinical remission, endoscopic healing, or prevention of relapse in patients with UC is currently insufficient. The available clinical literature should therefore be interpreted primarily as preliminary translational evidence supporting further investigation rather than as evidence for established therapeutic efficacy [12,17].

An important consideration is that pectin should not be regarded as a direct anti-inflammatory agent comparable to corticosteroids, biologic therapies, or small-molecule anti-inflammatory drugs. Rather, its potential therapeutic value may lie primarily in its ability to modify the intestinal metabolic environment, enhance epithelial resilience, and support conditions that are favorable for mucosal homeostasis and, potentially, mucosal healing. This concept is consistent with the emerging paradigm of adjunctive microbiome-directed therapy, in which nutritional and metabolic interventions are intended to complement, rather than replace, established pharmacological treatment [12]. Accordingly, the potential role of pectin in UC should be considered primarily as an adjunctive metabolic strategy rather than as an alternative to conventional anti-inflammatory therapy.

Several challenges currently limit the clinical translation of pectin-based interventions. Existing studies differ substantially with respect to pectin source, molecular weight, degree of methyl esterification, dosage, treatment duration, dietary background, concomitant medications, and methods used to characterize the intestinal microbiome [45,52,53,54]. Such heterogeneity makes direct comparison among studies difficult and limits the ability to determine whether specific structural characteristics of pectin are associated with greater biological or clinical efficacy. In addition, most available human studies have focused primarily on mechanistic or surrogate outcomes rather than clinically established endpoints such as sustained steroid-free remission, endoscopic healing, histological remission, or prevention of relapse. Another important consideration is the marked inter-individual variability in intestinal microbial composition and metabolic capacity. Because the biological effects of pectin depend, at least in part, on the presence and functional activity of microbial communities capable of degrading pectic polysaccharides and generating downstream metabolites, therapeutic responses are unlikely to be uniform across patients. Individuals with profound depletion of relevant microbial functions may therefore have a reduced capacity to respond to pectin supplementation. These considerations provide a rationale for future precision-nutrition approaches in which microbial and metabolomic characteristics are incorporated into patient selection and treatment stratification [24,25,26,27,29].

Future clinical research should therefore move beyond conventional dietary supplementation studies and adopt rigorously designed randomized controlled trials incorporating mechanistic and multi-omics endpoints. Simultaneous assessment of metagenomics, metatranscriptomics, metabolomics, and relevant host immune and epithelial biomarkers may help identify predictors of therapeutic response and clarify the biological pathways through which pectin may influence disease activity. Standardization of pectin preparations—including botanical source, molecular weight, degree of methyl esterification, structural composition, and dosage—will be essential to allow for meaningful comparison among clinical studies and to determine whether specific physicochemical characteristics are associated with greater biological activity. Dose-finding studies and longer treatment periods are also required to establish the optimal therapeutic exposure and to assess the durability of metabolic and clinical effects [14,15,38].

An additional area of considerable interest is the evaluation of pectin as an adjunct to established medical therapy. The proposed mechanism does not imply replacement of anti-inflammatory or immunomodulatory treatment; rather, pectin may potentially complement pharmacological therapy by modifying the intestinal metabolic environment, increasing endogenous SCFA availability, and improving epithelial barrier resilience. Combination strategies integrating microbiome-directed metabolic interventions with mesalamine, biologic agents, Janus kinase inhibitors, or other advanced therapies therefore represent a rational hypothesis for future investigation. Whether such an approach can improve clinical response, facilitate mucosal healing, reduce treatment-related inflammatory burden, or contribute to maintenance of remission remains unknown and should be determined prospectively in adequately powered clinical trials [1,2,61].

We can conclude that the available evidence indicates that pectin possesses substantial biological plausibility as a microbiome-directed metabolic intervention in UC. Although current human data remain insufficient to support formal therapeutic recommendations, the convergence of experimental, mechanistic, microbiological, and early clinical findings strongly justifies further translational investigation. Future studies should focus not only on demonstrating clinical efficacy but also on defining the patient populations, disease phenotypes, and microbial signatures most likely to benefit from pectin-based interventions.

Table 5 shows opportunities and challenges for the clinical translation of pectin therapy in UC.

## 5. Kaolin

### 5.1. Adsorbent Strategies: An Underexplored Field

While adsorbent therapy has received relatively little attention in contemporary inflammatory bowel disease research, experimental evidence suggests that luminal adsorption may influence intestinal inflammation through mechanisms distinct from direct immunomodulation. Oral adsorbents have been investigated primarily for their capacity to bind endotoxin and other potentially injurious luminal substances, thereby reducing their interaction with the intestinal mucosa. In experimental colitis, Gardiner et al. demonstrated that an adsorbent approach could reduce endotoxemia and attenuate mucosal injury, providing an early experimental basis for considering luminal adsorption as a potential adjunctive strategy in intestinal inflammation [11]. Importantly, these findings do not establish kaolin as an anti-inflammatory agent in the conventional pharmacological sense. Rather, they support the more cautious hypothesis that adsorption of luminal pro-inflammatory substances may reduce upstream stimuli capable of contributing to epithelial injury and innate immune activation. This distinction is particularly relevant when considering kaolin in combination with pectin: pectin may primarily act by restoring microbial fermentation and endogenous SCFA production, whereas kaolin could theoretically complement this metabolic effect by reducing the luminal burden of potentially injurious microbial products [9,11]. Such a complementary mechanism remains hypothetical and requires direct experimental and clinical validation.

### 5.2. Potential Limitations and Safety Considerations of Kaolin

Patients with UC may exhibit increased intestinal permeability and impaired mucosal barrier function, facilitating translocation of luminal bacterial products, including endotoxin, into the systemic circulation. In an experimental model of colitis, enteral administration of kaolin significantly reduced circulating endotoxin levels, providing evidence that mineral adsorbents can attenuate gut-derived endotoxemia by binding luminal pro-inflammatory bacterial products. Importantly, however, this effect should not be interpreted as evidence of a direct therapeutic effect on colonic inflammation. In the study by Gardiner et al., kaolin reduced systemic endotoxemia but did not significantly ameliorate colitis or restore mucosal integrity, and it did not increase colonic SCFA concentrations [11].

Thus, the potential role of kaolin in UC differs fundamentally from that of pectin: whereas pectin is hypothesized to restore endogenous SCFA production and thereby improve epithelial metabolic and barrier function, kaolin may act upstream by reducing the luminal availability and systemic translocation of potentially injurious bacterial products. The combination of these complementary mechanisms is therefore biologically plausible; however, this proposed dual action remains hypothetical and has not been evaluated in controlled clinical studies in patients with UC. Accordingly, the potential contribution of kaolin to the proposed pectin–kaolin therapeutic strategy should be regarded as a mechanistic hypothesis requiring dedicated experimental and clinical validation rather than as an established therapeutic effect.

An additional consideration concerns the non-selective adsorptive properties of mineral-based agents [18]. Experimental evidence indicates that kaolin-containing adsorbent preparations may alter the intestinal handling of luminal substances, including electrolytes and nutrients, although these observations derive primarily from experimental or non-IBD settings. Accordingly, kaolin may theoretically interact not only with luminal bacterial products but also with orally administered medications, bile acids, micronutrients, or other biologically active compounds. However, direct evidence demonstrating clinically relevant adsorption of mesalamine or other standard UC medications by kaolin is currently lacking. This possibility should therefore be regarded as a theoretical safety concern rather than an established pharmacological interaction and warrants formal in vitro binding studies followed, if appropriate, by pharmacokinetic studies in humans. Similarly, excessive or non-selective adsorption of luminal substrates could potentially modify the intestinal microbial environment and thereby influence microbial metabolic activity, although the direction and clinical significance of such effects remain unknown. These uncertainties are particularly relevant if kaolin is considered for prolonged adjunctive administration and should be specifically addressed in future translational studies [62,63,64]. Historical clinical studies of kaolin-containing preparations as antidiarrheal agents provide little additional evidence regarding their role in inflammatory bowel disease and cannot be extrapolated to the treatment of UC [19].

In summary, although kaolin has been used historically as an intestinal adsorbent, contemporary human evidence supporting its use in UC is virtually absent. Existing evidence is limited to experimental models demonstrating reduction in gut-derived endotoxemia and older clinical studies evaluating symptomatic diarrhea rather than IBD. Consequently, kaolin should currently be regarded as a biologically plausible adjunctive strategy requiring rigorous pharmacokinetic, mechanistic and randomized clinical evaluation before any therapeutic recommendations can be made [11,18,19,62,63,64].

Table 6 summarizes the available evidence regarding kaolin as a potential adjunctive therapy in UC.

### 5.3. Pectin–Kaolin Combination: A Dual-Mechanism Therapeutic Hypothesis

The complementary biological properties of pectin and kaolin provide the basis for a distinct, hypothesis-generating therapeutic concept in ulcerative colitis. Rather than targeting inflammation directly, the proposed combination is intended to address two potentially interconnected upstream components of intestinal injury: impaired microbial metabolic function and increased exposure of the mucosa to luminal injurious products. Pectin and kaolin therefore represent mechanistically different, but potentially complementary, interventions within the intestinal lumen.

Pectin may contribute primarily through restoration of endogenous microbial metabolism. As discussed in the preceding sections, pectin reaches the colon largely undigested and provides a fermentable substrate for the intestinal microbiota, thereby supporting microbial cross-feeding and endogenous production of SCFAs, particularly butyrate. Increased availability of SCFAs may improve colonocyte bioenergetics, reinforce epithelial barrier integrity, and promote a mucosal environment compatible with tissue repair and immune homeostasis. Importantly, these effects remain biologically plausible rather than clinically established in UC, and the magnitude of the response is likely to depend on the baseline functional capacity of the patient’s microbiome [16,56].

Kaolin, in contrast, would be expected to act primarily within the intestinal lumen through its adsorptive properties. Experimental evidence indicates that mineral adsorbents can reduce systemic exposure to gut-derived endotoxin in experimental colitis. The proposed mechanism is therefore not direct inhibition of inflammatory signaling but reduction in the luminal burden of potentially injurious bacterial products and their subsequent interaction with the intestinal mucosa. In this model, kaolin could theoretically reduce exposure to endotoxins and other adsorbable compounds, thereby decreasing downstream activation of pathways such as TLR4-dependent inflammatory signaling. However, this mechanism remains incompletely characterized, and direct evidence demonstrating that kaolin produces these effects in patients with UC is currently unavailable [11].

**Figure 7:**
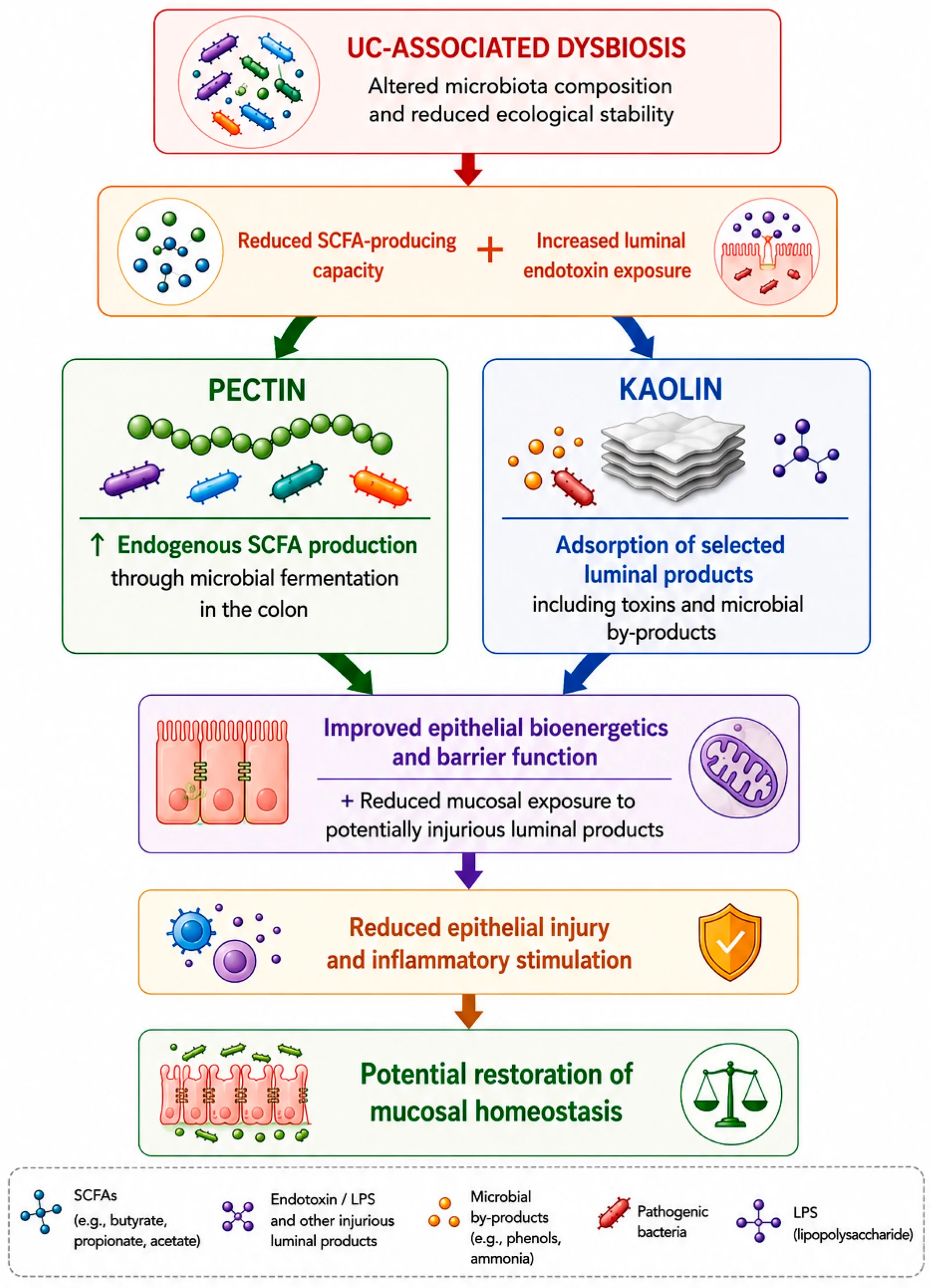
**Conceptual model of the proposed complementary effects of pectin and kaolin on intestinal homeostasis in ulcerative colitis.** Dysbiosis may be associated with reduced microbial SCFA-producing capacity and increased exposure of the intestinal mucosa to potentially injurious luminal products. Pectin may enhance endogenous SCFA production through microbial fermentation, whereas kaolin may adsorb selected luminal products, thereby potentially reducing their contact with the intestinal epithelium. The proposed downstream effects include improved epithelial bioenergetics and barrier function and reduced epithelial injury and inflammatory stimulation. The arrows represent a proposed mechanistic sequence based on biological plausibility and experimental evidence and should not be interpreted as demonstrating direct causal relationships in patients with UC.

The combination of these two mechanisms leads to a dual-compartment therapeutic hypothesis. Pectin would act predominantly on the microbial–metabolic compartment, promoting endogenous SCFA generation and restoration of epithelial metabolic competence, whereas kaolin would act predominantly on the luminal compartment, reducing exposure to selected potentially harmful microbial products. The two mechanisms could therefore converge at the level of the epithelial barrier: pectin may strengthen epithelial resilience from the metabolic side, while kaolin may reduce luminal injurious pressure from the intestinal side. The proposed complementary model linking dysbiosis, impaired SCFA production and increased luminal exposure to potentially injurious products, with the distinct but potentially convergent actions of pectin and kaolin, is summarized in Figure 7.

This model should, however, be interpreted strictly as a biological hypothesis rather than an established therapeutic mechanism. There is currently no clinical evidence demonstrating that pectin and kaolin act synergistically in UC, nor has the combination been adequately evaluated in experimental models specifically designed to distinguish additive or synergistic effects from the independent actions of either component. Moreover, kaolin is a non-selective adsorbent, and its potential effects on concomitantly administered medications, bile acids, micronutrients, and other luminal substrates require careful investigation. Similarly, the response to pectin may vary according to baseline microbial composition and fermentative capacity [11,18,19,62,63,64].

The principal scientific value of the proposed combination therefore lies in its testability. Experimental studies should first determine whether concurrent administration of pectin and kaolin produces greater improvements in epithelial integrity, inflammatory activity, endotoxemia, microbial composition, and SCFA production than either intervention alone. Pharmacokinetic studies should simultaneously establish whether kaolin alters the intestinal availability of standard UC medications, particularly orally administered mesalamine. If these preclinical studies demonstrate biological compatibility and a favorable safety profile, randomized clinical trials could subsequently evaluate the combination as an adjunct to established maintenance therapy.

Accordingly, pectin–kaolin therapy should presently be viewed not as an alternative to established anti-inflammatory or immunomodulatory treatment, but as a potential adjunctive metabolic–luminal intervention designed to complement pharmacological control of mucosal inflammation. This distinction is essential because the proposed strategy addresses mechanisms that are upstream or complementary to conventional immune-targeted treatment rather than replacing established therapies.

The dual-mechanism hypothesis also provides a conceptual bridge between the experimental evidence reviewed in the preceding sections and the future translational research agenda. Its ultimate validity will depend on prospective demonstration that restoration of microbial SCFA metabolism together with reduction in luminal injurious exposure can produce clinically meaningful improvements in sustained steroid-free remission, endoscopic and histological healing, and prevention of relapse. Until such evidence becomes available, the pectin–kaolin concept should remain explicitly hypothesis-generating.

## 6. Future Perspectives and Research Priorities

The rapidly expanding understanding of host–microbiome metabolic interactions is reshaping current concepts regarding the pathogenesis and treatment of UC. Increasing evidence suggests that restoration of microbial metabolic function may represent an important therapeutic objective that complements, rather than replaces, conventional immune-targeted therapies [9,10,20,23]. Within this evolving framework, pectin emerges as a promising microbiome-directed metabolic intervention whose potential extends beyond its conventional role as a dietary fiber. However, the current evidence remains predominantly preclinical, and its potential therapeutic value in UC requires confirmation in appropriately designed clinical studies [20,22,32,41].

A major priority for future research is the conduct of adequately powered, multicenter randomized controlled trials evaluating pectin as an adjunctive intervention in patients with UC. Such studies should employ standardized and well-characterized pectin formulations, with detailed specification of structural properties including degree of methyl esterification, molecular weight, botanical origin, and fermentation characteristics. Uniformly defined clinical endpoints—including clinical remission, endoscopic improvement or healing, histological activity, fecal calprotectin, quality of life, and steroid-free remission—will be essential to facilitate comparison among studies and enable meaningful evidence synthesis. Long-term studies should additionally evaluate maintenance of remission, treatment adherence, tolerability, and potential interactions with concomitant medications [45,52,53,54].

Future clinical trials should also integrate multi-omics technologies to characterize the biological effects of pectin across multiple levels of the host–microbiome interface. Combined metagenomic, metatranscriptomic, metabolomic, and, where appropriate, proteomic analyses may identify microbial functional signatures associated with treatment response while clarifying the metabolic pathways underlying potential clinical benefit. Integration of these datasets with host immune and epithelial phenotyping may facilitate the identification of candidate biomarkers capable of predicting response and guiding subsequent individualized interventions [24,25,26,27,29].

An equally important research direction involves the development of precision nutrition strategies tailored to individual microbiome composition and metabolic capacity. Therapeutic efficacy is likely to depend, at least in part, on the abundance and functional competence of pectinolytic and butyrate-producing microorganisms, as well as on the integrity of microbial cross-feeding networks. Baseline microbial and metabolic profiling may therefore permit stratification of patients according to their capacity to utilize pectin and generate beneficial metabolites [24,25,26,27,29]. Such an approach could eventually allow for microbiome-informed patient selection rather than empirical administration of the same pectin formulation to all patients. This hypothesis, however, requires prospective validation.

Optimization and standardization of pectin itself represent another important translational objective. Structural characteristics such as degree of methyl esterification, molecular weight, branching architecture, neutral sugar composition, and botanical source may influence microbial fermentation kinetics, SCFA production, and biological activity. Future comparative studies should determine whether specific structural profiles or formulations provide superior metabolic or clinical effects. Advances in food technology and pharmaceutical sciences may also facilitate the development of standardized, reproducible, and clinically appropriate pectin preparations with defined physicochemical and fermentation characteristics [45,52,53,54].

Combination strategies constitute another promising area of investigation. Because pectin is hypothesized to act primarily by modifying microbial metabolism, epithelial resilience, and the luminal environment rather than by directly suppressing immune activation, its most appropriate clinical role may be as an adjunct to established UC therapies. Mesalamine, corticosteroids, immunomodulators, biologic agents, and small-molecule therapies could therefore be evaluated in combination with pectin in prospective studies. Potential benefits may include improved mucosal resilience, enhancement of treatment-associated healing, or reduction in relapse risk; however, these possibilities remain hypothetical and should not be interpreted as evidence of therapeutic synergy until formally demonstrated in controlled clinical trials. Particular attention should also be given to potential pharmacokinetic interactions, especially with orally administered medications, given the adsorptive properties of kaolin when pectin and kaolin are considered as a combined formulation [62,63,64].

Emerging technologies, including computational systems biology and artificial intelligence, may play an increasingly important role in the development of microbiome-directed therapeutics. Machine-learning approaches integrating clinical, dietary, microbiological, metabolomic, and immunological variables could potentially identify patient subgroups with distinct metabolic phenotypes, predict treatment response, and assist in optimizing individualized nutritional interventions. Such approaches should, however, be regarded as tools for hypothesis generation and patient stratification until validated prospectively in independent clinical cohorts [24,25,26,27,29].

Beyond UC, the biological principles discussed in this review may have implications for other disorders characterized by disturbances in host–microbiome metabolic interactions, including Crohn’s disease, pouchitis, and other gastrointestinal conditions in which microbial metabolites and epithelial metabolism may contribute to disease expression. Investigation of pectin in these settings may help determine whether restoration of microbial metabolic function represents a broader therapeutic principle. Nevertheless, extrapolation from UC or experimental models to other diseases should be avoided until disease-specific evidence becomes available.

Ultimately, future therapeutic strategies are likely to move beyond the traditional distinction between pharmacological and nutritional interventions. Integrated approaches combining immune modulation, dietary intervention, microbiome-directed strategies, and metabolic restoration may provide a more comprehensive framework for long-term disease control. Within this evolving paradigm, pectin may serve as a prototype microbiome-directed metabolic intervention whose therapeutic potential remains to be established through rigorous translational research. The central objective should therefore not be simply to increase luminal SCFA concentrations, but to determine whether restoration of microbial metabolic function can produce measurable and clinically meaningful improvements in disease control.

**Figure 8:**
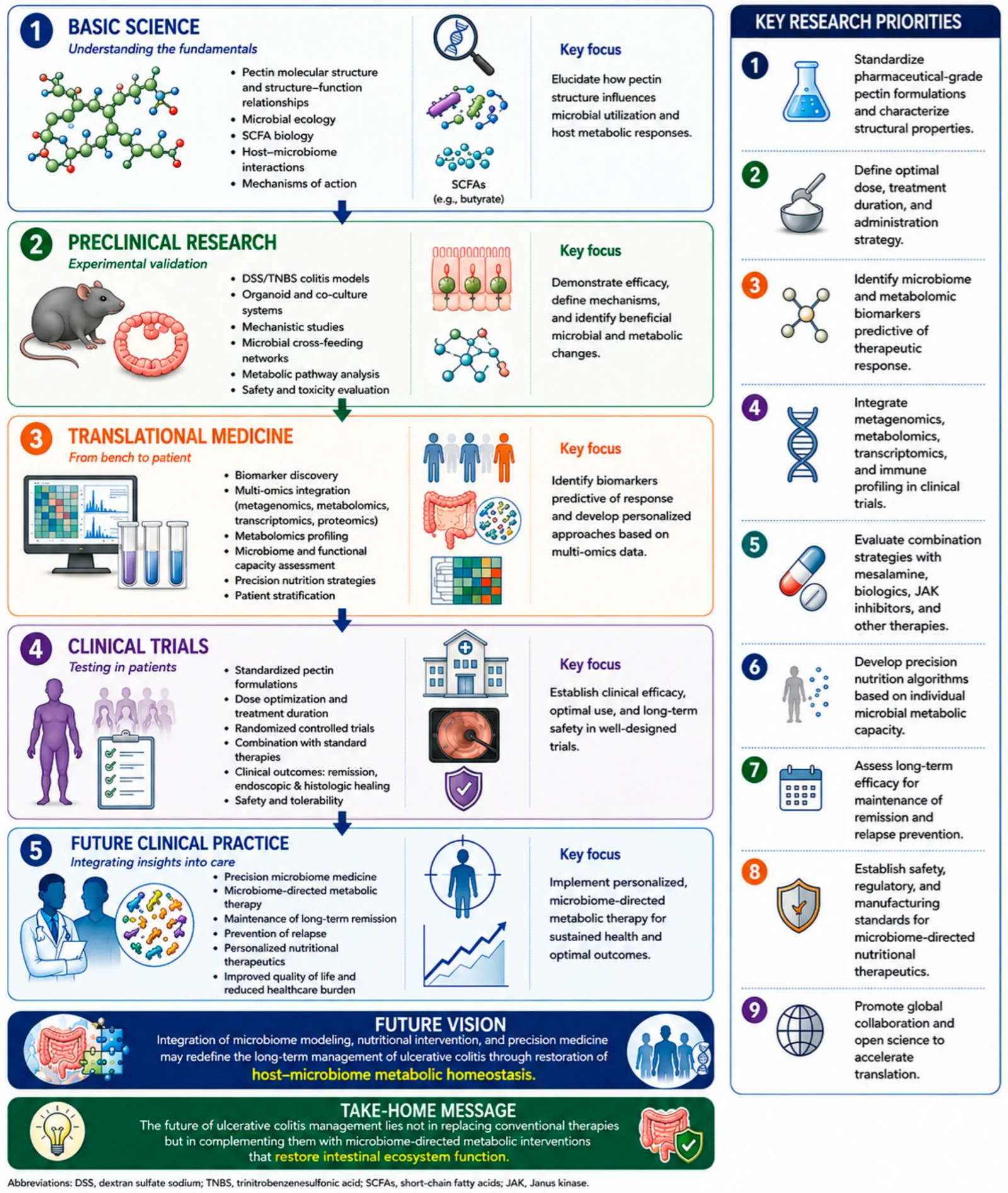
Roadmap toward microbiome-directed metabolic therapy in UC: from mechanistic discovery to precision clinical implementation. The table roadmap illustrates the proposed translational pathway for the development of pectin-based microbiome-directed metabolic therapy in UC. The process begins with basic scientific investigation aimed at defining the structure–function relationships of pectin, microbial ecology, SCFA biology, and host–microbiome metabolic interactions [45,52,53,54]. These mechanistic insights provide the foundation for preclinical studies using experimental colitis models, organoid systems, and advanced mechanistic analyses to characterize microbial cross-feeding networks, epithelial metabolic responses, and immunological pathways. The next phase focuses on translational medicine, integrating metagenomics, metatranscriptomics, metabolomics, proteomics, and immune profiling to identify predictive biomarkers of therapeutic response and establish the basis for precision nutrition strategies. These advances support the design of well-powered randomized clinical trials evaluating standardized pectin formulations, optimal dosing regimens, treatment duration, combination strategies with established ulcerative colitis therapies, and clinically meaningful endpoints, including endoscopic healing, histological remission, and long-term maintenance of remission. The ultimate objective is the implementation of personalized microbiome-directed metabolic therapy, in which nutritional interventions are tailored according to individual microbial composition, metabolic capacity, and host biological characteristics [24,25,26,27,29]. Such an approach has the potential to complement conventional pharmacological therapies by restoring microbial ecosystem function, epithelial bioenergetics, barrier integrity, and immune homeostasis rather than targeting isolated inflammatory pathways. The panel of Key Research Priorities highlights the principal scientific and clinical challenges that must be addressed before widespread implementation, including formulation standardization, optimization of dose and treatment duration, biomarker discovery, integration of multi-omics technologies into clinical trials, evaluation of combination therapies, development of precision nutrition algorithms, assessment of long-term efficacy and safety, and establishment of regulatory standards for microbiome-directed nutritional therapeutics. Overall, the roadmap emphasizes a paradigm shift from symptom-oriented treatment toward restoration of host–microbiome metabolic homeostasis through integrated nutritional, microbial, metabolic, and immunological interventions. Within this emerging framework, pectin represents a prototype microbiome-directed metabolic regulator whose therapeutic potential should be validated through rigorous translational and clinical research before incorporation into routine management of UC [33,35,36].

Figure 8 summarizes a proposed translational roadmap for the development and clinical evaluation of pectin-based microbiome-directed metabolic therapy in UC, progressing from mechanistic and preclinical evidence through formulation standardization, biomarker discovery, clinical trial evaluation, and ultimately to evidence-based patient selection and integration with established UC therapies.

## 7. Conclusions

UC is increasingly recognized as a disorder in which chronic immune activation is closely interconnected with disturbances in microbial ecology, metabolite production, and epithelial metabolic function. Although current therapeutic strategies have substantially improved disease control by targeting inflammatory pathways, they do not directly correct the metabolic consequences of dysbiosis and depletion or functional impairment of SCFA-producing microorganisms. Restoration of host–microbiome metabolic homeostasis therefore represents an emerging therapeutic objective that may complement, rather than replace, established pharmacological approaches [9,10,20,23].

Among microbiome-directed nutritional interventions, pectin possesses several characteristics that distinguish it from conventional dietary fibers. Its complex molecular architecture and fermentability allow for microbial utilization and may support reconstruction of microbial cross-feeding networks and endogenous production of SCFAs, particularly butyrate. Beyond serving as a fermentable substrate, pectin may influence epithelial bioenergetics, intestinal barrier integrity, oxidative stress, immune regulation, and microbial ecological stability. These potentially interconnected actions support a systems-level model of intestinal homeostasis rather than an isolated anti-inflammatory effect [45,52,53,54,55]. Experimental studies provide substantial evidence that pectin can attenuate intestinal inflammation, reinforce epithelial barrier function, modify microbial metabolism, reduce oxidative injury, and promote mucosal repair in several models of experimental colitis [16,44,45,54,57,58,59,60]. In contrast, human evidence remains limited and heterogeneous. Available clinical observations provide preliminary indications of favorable effects on selected microbial, metabolic, and inflammatory parameters, but they are insufficient to establish clinical efficacy or to support routine therapeutic use. Accordingly, the biological rationale for pectin is considerably stronger than the current clinical evidence [16,56].

Future progress will depend on the integration of nutritional science, microbiome research, metabolomics, immunology, and precision medicine. Well-designed, adequately powered randomized controlled trials using rigorously characterized and standardized pectin preparations will be required to determine optimal dose, formulation, treatment duration, and patient selection. Comprehensive multi-omics characterization may help identify microbial and metabolic signatures associated with treatment response and clarify the mechanisms underlying potential clinical benefit. Such studies should also determine whether pectin can provide clinically meaningful additional benefit when combined with established therapies, including mesalamine, biologic agents, and small-molecule therapies. Importantly, future research should incorporate clinically relevant outcomes such as steroid-free clinical remission, endoscopic and histological healing, fecal calprotectin normalization, quality of life, and prevention of relapse rather than relying predominantly on surrogate biomarkers.

The evidence synthesized in this review supports a conceptual shift in the interpretation of pectin. Rather than being regarded solely as a soluble dietary fiber or conventional prebiotic, pectin may be viewed as a potential microbiome-directed metabolic intervention capable of influencing several interconnected components of intestinal physiology, including microbial ecology, SCFA metabolism, epithelial function, barrier integrity, oxidative stress, and immune homeostasis. This systems-based perspective provides a coherent framework for integrating the diverse experimental observations currently available while recognizing the substantial uncertainties that remain regarding structure–function relationships, microbial responsiveness, optimal formulation, and clinical efficacy [45,52,53,54,55,56,57,58].

Ultimately, the future management of UC may extend beyond exclusive modulation of host inflammatory pathways toward integrated strategies that simultaneously address immune dysregulation, microbial ecology, and intestinal metabolic function. Within this emerging framework, pectin represents a biologically plausible candidate for adjunctive microbiome-directed metabolic therapy, but its clinical role remains to be established. The convergence of mechanistic and experimental evidence provides a strong rationale for continued translational investigation; however, the limited human evidence currently precludes therapeutic recommendations. Definitive evaluation will require rigorous randomized clinical trials capable of determining whether restoration of pectin-mediated microbial metabolic function can translate into sustained improvements in mucosal healing, remission maintenance, and long-term outcomes in patients with UC [12,17,52,53].

## Figures and Tables

**Table 1:** Metabolites, producing microbes and effects in UC.

Metabolite (Alteration in UC)	Microbes That Produce/Modify It (Examples)	Reported Effect on Host/Relevance in UC
Butyrate (decreased)	*Faecalibacterium prausnitzii*, *Roseburia* spp., *Eubacterium rectale*/*Eubacterium hallii* [butyrate] [10,20]	Energy source for colonocytes, promotes barrier integrity and anti-inflammatory signaling; reduced levels associated with mucosal injury and active UC [10,20]
Other SCFAs (acetate, propionate) (often reduced/imbalanced)	*Bacteroides* spp., *Prevotella*, many Firmicutes species [8,25]	SCFAs regulate immune responses and epithelial health; altered ratios contribute to dysregulated mucosal immunity in UC [10,20]
Succinate (elevated) [10]	Bacteroidetes and certain Prevotella/Bacteroides/commensals producing succinate as intermediate [9]	Acts as a pro-inflammatory signal activating immune cells and promoting colitis; elevated succinate linked to active UC [10]
Secondary bile acids (altered: often decreased protective secondary BAs, altered composition) [8,9,20,28]	Clostridium cluster XIVa (e.g., *Clostridium* spp.), Bacteroides, other BA-modifiers (7α-dehydroxylating bacteria) [8,9,20,28]	Secondary bile acids modulate epithelial and immune signaling; disrupted bile acid metabolism correlates with inflammation and FMT response in UC [8,9,20,28]
Tryptophan metabolites (indoles; decreased)	*Lactobacillus*, *Bacteroides*, *Clostridium* species that metabolize tryptophan to indoles [8,9,20,28]	Indole derivatives activate the aryl hydrocarbon receptor (AhR) and support mucosal immunity; loss contributes to barrier dysfunction and inflammation [8,9,20,28]
Lipid metabolites/altered lipidome (dysregulated) [23,24]	Microbial actions (Bacteroides, other Gram-negatives) and host–microbe interactions; specific taxa vary by study [21,28]	Altered lipid metabolism in epithelial cells and fecal lipid profiles associated with epithelial dysfunction and UC pathogenesis
Heme and heme-related metabolites (elevated in non-responders) [23,24]	*Proteobacteria* (Escherichia), some Fusobacterium species (associated with heme metabolism pathways) [10,11]	Heme-derived metabolites can promote oxidative stress and pathogenic growth; associated with poor FMT response and active disease [21,28]
Lipopolysaccharide (LPS) biosynthesis (increased signatures)	Gram-negative taxa (Escherichia, some Bacteroides)	Elevated LPS-related pathways promote innate immune activation and mucosal inflammation in UC [24]
Bacterial quorum-sensing and other small molecules (altered)	Diverse taxa; specifics still under study (PubMed). [23]	Can modulate microbial community behavior and host signaling; implicated in pathogenesis but mechanistic details are evolving

**Table 2:** SCFA-mediated molecular mechanisms.

Mechanism	SCFAs	Molecular Target/Pathway	UC-Relevant Effect	Evidence in UC Context
Epithelial uptake and metabolic use	Butyrate > acetate, propionate	MCT1/SLC16A1, SMCT1/SLC5A8, SMCT2; butyrate oxidation enzymes	Inflamed UC mucosa shows reduced transporter expression, reduced butyrate uptake/oxidation, and downregulated oxidative genes, which would limit epithelial access to SCFAs [33]	Carrier-mediated uptake is the major epithelial entry route for SCFAs, and transporter expression/function is altered in UC [31]
Barrier reinforcement	Butyrate, acetate	Tight-junction programs, HIF1α, colonocyte oxygen consumption, epithelial resistance	SCFAs support barrier integrity by fueling colonocytes and strengthening junctional programs; acetate increased epithelial resistance and upregulated HIF1α and MUC2 in UC patient-derived monolayers [39]	Butyrate increases epithelial oxygen consumption and stabilizes physiological hypoxia–HIF signaling that supports barrier function [21]
Mucus barrier repair	Butyrate	Macrophage/WNT/ERK axis; goblet-cell programs MUC2 and SPDEF	Butyrate increased mucin production and goblet-cell abundance through macrophage-dependent signaling, with WNT secretion and ERK1/2 activation required for the macrophage-mediated effect [16]	In DSS injury, transfer of butyrate-induced M2 macrophages restored goblet cells and mucus [16]
GPCR-mediated anti-inflammatory signaling	Acetate, propionate, butyrate, valerate	GPR41/FFAR3GPR43/FFAR2, GPR109A; downstream AKT/NF-κB, IL-18	SCFAs signal through GPCRs to suppress inflammatory pathways; butyrate–GPR109A signaling promotes epithelial IL-18 and colitis resistance, while valerate suppresses macrophage activation through GPR41/GPR43 upregulation [33]	Reviews and disease-focused syntheses consistently identify GPCR signaling as a core mechanism in IBD/UC [20,35]
HDAC inhibition and transcriptional control	Butyrate, propionate, acetate	Histone deacetylases; histone H3 acetylation; Foxp3 locus	Intracellular SCFA actions include HDAC inhibition, which links SCFAs to anti-inflammatory gene programs; butyrate induces Treg differentiation by increasing histone H3 acetylation at Foxp3-associated regions [28]	HDAC inhibition also appears to mediate barrier protection, because pharmacologic HDAC inhibitors mimicked butyrate in restoring cytokine-disrupted epithelial integrity [20]
Treg induction	Butyrate, mixed SCFAs	Foxp3 induction; GPR43 on T cells	SCFAs promote regulatory T-cell polarization in colitis models, including butyrate-driven Foxp3 acetylation and GPR43-linked Treg induction [11]	This mechanism is a leading explanation for SCFA-mediated mucosal immune tolerance in colitis https://consensus.app/papers/butyrate-and-mucosal-inflammation-new-scientific/b19af53399da5ecf89626519425ab25b/ (accessed on 19 August 2026).
Macrophage polarization	Butyrate, valerate, mixed SCFAs	M2 polarization; JAK/STAT3/FOXO3 inactivation; IL-6 suppression	Butyrate promotes M2 macrophage polarization with increased CD206 and Arg1, and SCFA-producing strains improve DSS colitis by inducing M2 macrophages that inhibit the JAK/STAT3/FOXO3 axis [37]	Valerate also suppresses activated macrophages and improves murine colitis, linking noncanonical SCFAs to macrophage-centered UC mechanisms [35]
Suppression of innate inflammatory signaling	Acetate, propionate, butyrate	NF-κB p65, MAPK, spleen tyrosine kinase, TLR-triggered cytokine programs	Acetate and propionate suppress TNF-α-induced NF-κB/MAPK signaling, and SCFAs broadly reduce TLR-driven pro-inflammatory responses in epithelial and myeloid cells [14,28]	In a human UC physiomimetic model, SCFAs reduced innate immune activation partly through PPAR signaling and NF-κB downregulation [22]
Tight-junction and epithelial gene regulation	Acetate, butyrate, mixed SCFAs	ZO-1, occludin, claudin-1, epithelial activation pathways	SCFA-producing strains increased ZO-1, occludin, and claudin-1 in experimental colitis, while acetate improved epithelial resistance and reduced IL8 and TNFα in UC monolayers [15]	Butyrate provided the strongest prolonged protection against cytokine-induced epithelial permeability in coculture [21]
Microbe–epithelium signaling	Butyrate from Roseburia intestinalis	SP3→TLR5 transcriptional upregulation	Butyrate increased SP3 binding to the TLR5 promoter, raising epithelial TLR5 transcription and associating microbial butyrate production with anti-inflammatory signaling in colitis [40]	*R. intestinalis* also increased epithelial TLR5 transcription and improved murine colitis [40]
Context-dependent or paradoxical effects	Mixed SCFAs	Metabolic reprogramming of innate and effector CD4 T cells	SCFAs reduced innate immune activation in a human UC gut–liver–immune model, but during acute T-cell-mediated inflammation they increased effector CD4 activity, worsened barrier disruption, and increased hepatic injury [38]	This helps explain why therapeutic studies can show heterogeneous effects despite strong anti-inflammatory mechanisms [32,41]

**Table 3:** Characteristics of an ideal SCFA-restoring prebiotic.

Characteristic	Biological Significance
Selective fermentability	Preferentially utilized by beneficial commensal bacteria rather than opportunistic pathogens.
Promotion of butyrate-producing bacteria	Enriches taxa such as *Faecalibacterium*, *Roseburia*, *Anaerostipes*, *Eubacterium*, and other SCFA-producing microorganisms.
Restoration of microbial cross-feeding [20,33,34]	Supports cooperative metabolic interactions required for efficient endogenous butyrate synthesis.
Sustained SCFA generation	Increases physiological production of acetate, propionate, and particularly butyrate within the colon.
Preservation of epithelial energy metabolism	Restores colonocyte mitochondrial β-oxidation and ATP production through endogenous butyrate availability.
Improvement of intestinal barrier integrity	Enhances tight-junction protein expression, mucin secretion, and epithelial repair while reducing intestinal permeability [20,33,34].
Immunomodulatory activity	Promotes regulatory T-cell differentiation, suppresses NF-κB activation, and reduces production of pro-inflammatory cytokines.
Modulation of microbial metabolism beyond SCFAs	Favors beneficial bile acid transformation, tryptophan metabolism, and other metabolite networks involved in intestinal homeostasis.
High safety and tolerability	Suitable for long-term administration with minimal adverse effects.
Compatibility with standard UC therapy	Can be combined with mesalamine, corticosteroids, immunomodulators, biologics, or small molecules without significant drug interactions.
Potential for personalized nutrition [25,26,27]	Allows for adaptation according to the patient’s microbiome composition and metabolic profile.

**Table 4:** Principal biological effects of pectin in experimental models of UC.

Pathophysiological Domain	Effects of Pectin	Representative Experimental Findings
Gut microbiota [16,44,45,56]	↑ SCFA-producing bacteria, restoration of microbial diversity	↑ *Faecalibacterium*, *Roseburia*, *Anaerostipes*
SCFA production [16,44,45,56]	↑ Butyrate, acetate, propionate	Improved microbial fermentation
Epithelial metabolism	↑ Mitochondrial β-oxidation, ↑ ATP production	Improved colonocyte bioenergetics
Barrier integrity [16,45,57,58]	↑ ZO-1, occludin, claudin-1; ↑ mucin	Reduced intestinal permeability
Immune regulation	↓ TNF-α, IL-1β, IL-6, IL-17; ↑ IL-10, ↑ Treg	Reduced mucosal inflammation
Oxidative stress [16,54,60]	↑ Nrf2, ↑ SOD, GPx, CAT, HO-1	Reduced ROS and lipid peroxidation
Histopathology [16,45,54,57,58].	↓ Ulceration, ↓ inflammatory infiltrate	Improved histological scores
Clinical outcomes (animal models) [16,45,54,57,58]	↓ Disease activity index, ↓ weight loss, ↑ mucosal healing	Attenuation of DSS/TNBS colitis

↑ means increase, ↓ means decrease.

**Table 5:** Opportunities and challenges for the clinical translation of pectin therapy in UC.

Potential Advantage	Current Limitation	Future Research Priority
Restoration of endogenous SCFA production	Limited human clinical evidence [12,17]	Large, adequately powered multicenter RCTs
Improvement of epithelial barrier integrity	Heterogeneous pectin formulations [55,56,61,62]	Standardized, well-characterized pectin formulations
Modulation of gut microbiota [25,26,27]	Variable baseline microbiome composition	Precision microbiome-guided nutrition
Favorable short-term tolerability	Limited long-term efficacy and safety data [12,17]	Long-term maintenance and safety studies
Compatibility with current UC therapies	Limited combination studies [61].	Adjunctive trials with biologics and small molecules
Multi-target mechanism of action	No validated predictive biomarkers	Development and validation of multi-omics biomarkers

**Table 6:** Current evidence regarding kaolin as a potential adjunctive therapy in UC.

Domain	Available Evidence	Principal Findings	Level of Evidence	Major Limitations/Knowledge Gaps
Adsorption of bacterial toxins [11]	Experimental in vitro studies	Kaolin adsorbs bacterial toxins and other luminal compounds through non-selective surface interactions.	Moderate (experimental)	No direct demonstration in UC patients.
Adsorption of bile acids	Experimental studies	Kaolin may bind bile acids, potentially reducing bile acid-induced epithelial irritation.	Low– moderate	Clinical relevance in UC remains unknown.
Reduction in luminal inflammatory burden	Animal and experimental observations	Adsorptive properties may decrease exposure of the mucosa to injurious luminal factors.	Low	No human confirmation.
Effects on intestinal barrier integrity	Indirect mechanistic evidence	A reduction in luminal irritants could theoretically preserve epithelial barrier function.	Very low	No direct experimental confirmation.
Interaction with gut microbiota	No direct evidence	No studies demonstrate that kaolin modifies microbial composition or increases SCFA production.	Very low	Major knowledge gap.
Interaction with SCFA metabolism	No direct evidence	No evidence that kaolin directly enhances microbial fermentation or SCFA generation.	Very low	Hypothesis only.
Clinical studies in ulcerative colitis	None	No randomized or observational studies evaluating kaolin as adjunctive therapy for UC remission maintenance.	Very low	Complete absence of clinical evidence.
Potential drug interactions [40,63,64]	Theoretical pharmacological concern	Possible adsorption of orally administered drugs (e.g., mesalamine) should be considered.	Very low	Requires pharmacokinetic studies.
Potential nutrient interactions [18]	Theoretical concern	Possible adsorption of vitamins, trace elements or other nutrients during prolonged administration.	Very low	No dedicated human studies.
Overall assessment	Mechanistic hypothesis	Biological plausibility exists, but current evidence is insufficient to support clinical use.	Very low certainty	Randomized clinical trials and pharmacokinetic studies are required.

SCFAs, short-chain fatty acids; UC, ulcerative colitis. Evidence levels represent an overall qualitative assessment based on the current literature and are intended to summarize biological plausibility rather than formal grade recommendations.

## Data Availability

No new data were created or analyzed in this study. Data sharing is not applicable to this article.
